# Diet-sourced carbon-based nanoparticles induce lipid alterations in tissues of zebrafish (*Danio rerio*) with genomic hypermethylation changes in brain

**DOI:** 10.1093/mutage/gew050

**Published:** 2016-10-26

**Authors:** Eva Gorrochategui, Junyi Li, Nigel J. Fullwood, Guang-Guo Ying, Meiping Tian, Li Cui, Heqing Shen, Sílvia Lacorte, Romà Tauler, Francis L. Martin

**Affiliations:** ^1^Department of Environmental Chemistry, Institute of Environmental Assessment and Water Research (IDAEA), Consejo Superior de Investigaciones Científicas (CSIC), Barcelona, 08034, Catalonia, Spain,; ^2^Centre for Biophotonics and; ^3^Biomedical and Life Sciences Division, Lancaster Environment Centre, Lancaster University, Lancaster LA1 4YQ, UK,; ^4^State Key Laboratory of Organic Geochemistry, Guangzhou Institute of Geochemistry, Chinese Academy of Sciences, Guangzhou 510640, China,; ^5^Key Lab of Urban Environment and Health, Institute of Urban Environment, Chinese Academy of Sciences, Xiamen 361021, China and; ^6^Biosciences, School of Pharmacy and Biomedical Sciences, Maudland Building, University of Central Lancashire, Preston PR1 2HE, UK

## Abstract

With rising environmental levels of carbon-based nanoparticles (CBNs), there is an urgent need to develop an understanding of their biological effects in order to generate appropriate risk assessment strategies. Herein, we exposed zebrafish *via* their diet to one of four different CBNs: C_60_ fullerene (C_60_), single-walled carbon nanotubes (SWCNT), short multi-walled carbon nanotubes (MWCNTs) or long MWCNTs. Lipid alterations in male and female zebrafish were explored post-exposure in three target tissues (brain, gonads and gastrointestinal tract) using ‘omic’ procedures based in liquid chromatography coupled with mass spectrometry (LC-MS) data files. These tissues were chosen as they are often target tissues following environmental exposure. Marked alterations in lipid species are noted in all three tissues. To further explore CBN-induced brain alterations, Raman microspectroscopy analysis of lipid extracts was conducted. Marked lipid alterations are observed with males responding differently to females; in addition, there also appears to be consistent elevations in global genomic methylation. This latter observation is most profound in female zebrafish brain tissues post-exposure to short MWCNTs or SWCNTs (*P* < 0.05). This study demonstrates that even at low levels, CBNs are capable of inducing significant cellular and genomic modifications in a range of tissues. Such alterations could result in modified susceptibility to other influences such as environmental exposures, pathology and, in the case of brain, developmental alterations.

## Introduction

Carbon-based nanoparticles (CBNs) are key constituents in many technologies. As a consequence, environmental exposure is increasing in the absence of a full understanding of the potential risks that they might pose. Significant gaps in our understanding or the ability to assess such risks (1) exist and these include: (i) as they differ from chemicals, an absence of an understanding of their toxicodynamics and toxicokinetics; (ii) an obscure mechanism of action; (iii) an absence of identified endpoints; and (iv) a lack of assays of exposure and effect. There is an urgent need to determine CBN-induced environmental effects in target organisms and to develop approaches towards measuring endpoint outcomes (2,3).

**Figure 1.**
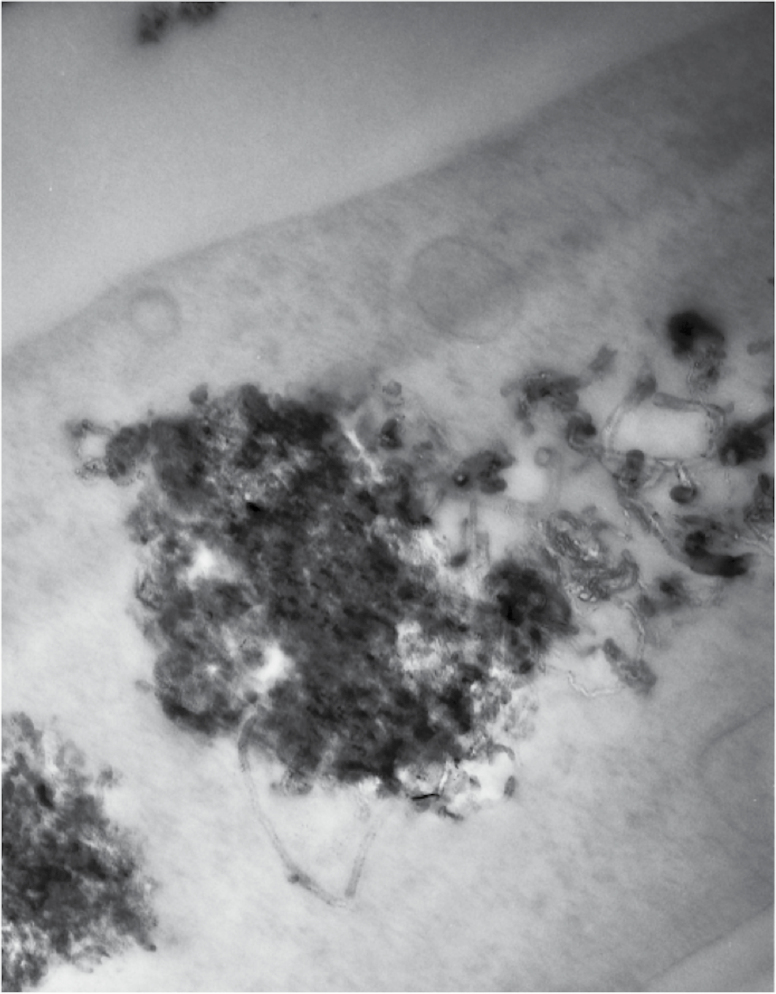
Transmission electron micrograph of a clump of nanotubes within the cytoplasm of a cell. Individual MWNTs are visible projecting from edges of the clump. The micrograph shows how large quantities of nanotubes can be internalised and accumulated within the cell cytoplasm.

CBNs comprise a diverse range of entities including multi-walled carbon nanotubes (MWCNTs; long or short), single-walled carbon nanotubes (SWCNTs) and C_60_ fullerene (C_60_). These carbon allotropes with cylindrical nanostructures have unusual properties that confer advantages in electronics, optics, materials sciences and nanotechnology. In the environment, they possess the ability to either interact with cell membranes or even penetrate into cells to interact with organelles. Figure 1 shows an example of long MWCNTs that have penetrated into a stromal fibroblast, taken from a representative exposed tissue. They may surround or further penetrate into subcellular structures such as lysosomes. The consequences of such effects might be the generation of reactive oxygen species (ROS), rupture of organelles with consequent leakage of degradative enzymes or effects on the lipidome (2,4). Understanding the effects on the lipidome is of crucial interest, because lipids play essential roles in energy production and storage and cell membrane development (5,6), and thus, their study can aid in understanding the pathogenesis of many disease states. Given the multiplicity of possible outcomes, ‘omic’ procedures might be a better approach towards determining endpoint effects. The significance of parameters such as lipid peroxidation, potential genetic damaged induced by ROS and genomic methylation are critical to our understanding of environmental nanotoxicology (7–12).

Omic procedures using liquid chromatography coupled with mass spectrometry (LC-MS)-based methods have the ability for the analysis of low-molecular-weight compounds, such as complex lipid mixtures or biomolecules, in biological systems (13). The large amount of information contained in LC-MS data files (14) can be processed using chemometric tools such as multivariate curve resolution–alternating least squares (MCR-ALS) (15), which has been shown in recent omic (e.g. lipidomic) studies to successfully resolve extensive LC-MS data sets with strongly co-eluted and hidden peaks (16–21). In addition, principal component analysis (PCA) or partial least squares discriminant analysis (PLS-DA) can further explore sample classification/discrimination and allow biomarker discovery (22).

Application of spectrochemical methods such as Raman microspectroscopy to biological systems follows a similar premise (23). The advantage of this reagent-free and non-destructive approach is that it readily generates a signature fingerprint of the analysed sample. Applying a computational framework to a generated data set allows one to extract distinguishing features associated with altered chemical bonds; these can be associated with constituents such as lipids, proteins or DNA/RNA (24). Raman microspectroscopy has been applied to shed insights into CBN-induced effects (25,26), cell lineage (27) and to generate tissue images (28).

Considering that ROS generation might be an underlying mechanism of CBN-induced toxicity, herein we examine the effects of low dietary exposures on three target tissues (brain, gonads and gastrointestinal tract) in male and female zebrafish. Although many such studies have examined liver, a major downside to this approach is that pathological effects rarely feature highly in this target tissue. Central to our hypothesis for conducting this study, we chose gastrointestinal tract as the initial target tissue following dietary exposure, brain as there is an emerging notion that nanoparticles might modify development and gonads to determine any potential effects on reproductive biology. Primarily, we examine lipid alterations through LC-MS and Raman microspectroscopy; this is extended to examine genomic methylation levels post-exposure in brain. Our aim is to examine whether sublethal CBN exposures might induce subtle alterations that could ultimately be associated with development or pathological changes.

## Animals, materials and methods

### Chemicals and CBNs

Bovine serum albumin (BSA) obtained from Sigma was ≥98%. All CBNs were purchased from Sigma. Short MWCNTs were >90% pure being 10–15 nm in diameter and 0.1–10 μm in length. Long MWCNTs were >90% pure also but were 110–170 nm in diameter and 5–9 μm in length. C_60_ had a purity >99.5% and particle size of 1 nm. SWCNTs were described as CarboLex AP-grade (the purity of AP-grade products ranges from 50% to 70% by volume); major impurities are carbon nanospheres and carbon-encapsulated catalyst nanoparticles—the diameter was 1.2–1.5 nm. All CBNs were analysed by Raman spectroscopy (Renishaw PLC, UK) with a 785 nm laser and determined to be of high purity; CBNs were reanalysed throughout the course of the study and no degradation in their purity was noted during this time. Additionally, images of CBNs were taken using a scanning electron microscope (JSM 5600, JEOL) (2–4). CBNs were dispersed in 1% BSA solution with a 15-min ultrasonication and stock solutions were made at concentrations of 100 mg L^−1^ and 1 mg L^−1^. CNT solutions were stable and well-dispersed, while C_60_ appeared to agglomerate.

Such CNT solutions are known to be stable (29). In DLVO theory, the agglomeration and stability of particle dispersions are determined by the sum of the attractive and repulsive forces between individual particles. The attraction between particles is due to van der Waals forces. The interaction of electrical double layers surrounding each particle is called electrostatic repulsive force. Typically, when dispersing CNPs in solution, they can form agglomerates, or remain as aggregates, surrounded by an electrical double layer. To overcome this problem, two different approaches are currently being used to disperse carbon nanotubes, i.e. mechanical (or physical) methods and chemical methods. Using mechanical methods such as ultrasonication with a suitable period of mixing, nanotubes are separated from each other. Chemical methods use surfactants or chemical moieties to change the surface energy of the nanotubes, improving their wetting or adhesion characteristics and improving their dispersion stability in solvent. However, many dispersants cannot be applied to biological research due to their intrinsic toxicity in *in vitro* and *in vivo* systems. The non-covalent adsorption of BSA, as a biocompatible dispersant, on the surface of CNTs could change the surface charge of CNTs. However, C_60_ is more likely to aggregate forming as *n*C_60_ than CNTs.

### Fish maintenance and experimental conditions

All experiments were carried out in accordance with local Institutional Review Board requirements. Zebrafish were maintained in the Aquatic Toxicology Laboratory at the Guangzhou Institute of Geochemistry (Chinese Academy of Sciences). All fish were kept in 50 L flow-through tanks filled with dechlorinated tap water in a temperature-controlled room maintained at 27 ± 1°C. The room was on a 14:10 h light:dark cycle, and fish were fed once daily with a quantity of commercial food at 5% of the wet weight.

Fish exposures were conducted in 10 L glass tanks, and each experimental tank contained 5 L dechlorinated tap water and 4 fish (2 males and 2 females). Prior to exposure, zebrafish in 50 L tanks were randomly transferred to the experimental tank for a 7-day adaptive period. Following this, CNP exposure was initiated and run for 21 days. There were 15 randomly assigned tanks for total exposure (control and treatment with each CBN at 0.1 mg L^−1^ in triplicate). Exposure concentration was chosen based on previous *in vitro* studies (2), which showed that, speculated real-world environmental levels induced alterations in exposed cell populations are detectable by biospectroscopy techniques (3, 4). To minimize contamination, fish were only fed in the morning every day; in the afternoon, each tank was cleaned to remove fish faeces and food remains by siphoning the water out of the tanks. Then all tanks were filled with fresh water and fresh treatment. All fish were killed within seconds by immersion in melting ice at the end of the exposure. From each fish, brain, gonads and intestine were independently harvested and stored in liquid nitrogen for further analysis. Exposure experiments were conducted in triplicate.

### Transmission electron microscopy

Stromal fibroblasts were isolated from a representative tissue section post-exposure to long MWCNTs; these were fixed in 2.5% glutaraldehyde in 0.1 M cacodylate buffer for 2 h. Samples were washed three times in buffer before being post fixed in 2% aqueous osmium tetroxide for 1 h. Samples were washed again in buffer prior to dehydration through a graded ethanol series. They were then transferred to propylene oxide for two 30-min steps and transferred to Araldite resin overnight before being placed in fresh resin and polymerised for 24 h at 60°C. Ultrathin sections were collected on copper grids stained briefly with phosphotungstic acid and Reynold’s lead citrate prior to examination on a JEOL 10-10 transmission electron microscope.

### Lipid extraction

Approximately 0.5 mg amounts of tissue were homogenized in 0.1% ammonium acetate. Then methanol (1.5 mL) was added to a 200-µL sample aliquot, which was placed into a glass tube with a Teflon-lined cap, and the tube was vortexed. Five millilitre of methyl tert-butyl ether (MTBE) was added and the mixture was incubated for 1 h at room temperature in a shaker. Adding 1.25 mL of high-performance liquid chromatography-grade water-induced phase separation. Upon 10 min of incubation at room temperature, the sample was centrifuged at 1000 *g* for 10 min. The upper (organic) phase was collected, and organic phases were dried in a vacuum centrifuge. Half of the extracted lipids were dissolved in MTBE for Surface-enhanced Raman spectroscopy (SERS), the other was dissolved in 200 µL of methanol for further LC-high–resolution mass spectrometry (HRMS) analysis.

### LC-MS analysis

LC-HRMS analysis was performed using an Acquity ultrahigh-performance liquid chromatography (UHPLC) system (Waters, USA) connected to a time-of-flight (LCT Premier XE) detector with an Acquity UHPLC BEH C_8_ column (1.7 µm particle size, 100 mm × 2.1 mm, Waters, Ireland) at a flow rate of 0.3 mL min^−1^ and column temperature of 30°C. Full scan spectra from 50 to 1500 Da were acquired, and individual spectra were summed to produce data points each of 0.2 s. Mass accuracy at a resolving power of 10 000 and reproducibility were maintained using an independent reference spray *via* the LockSpray interference. The mobile phases were methanol with 2 mM ammonium formiate and 0.2% formic acid (A)/water with 2 mM ammonium formiate and 0.2% formic acid (B). Phosphatidylcholine (PC), plasmalogen PC, lyso plasmalogen PC, phosphatidylethanolamine (PE), phosphatidylserine (PS), diacylglycerol (DAG) and triacylglycerol (TAG) species were analysed under positive ESI (electrospray ionization).

### Chemometric analysis of LC-HRMS data

**Figure 2.**
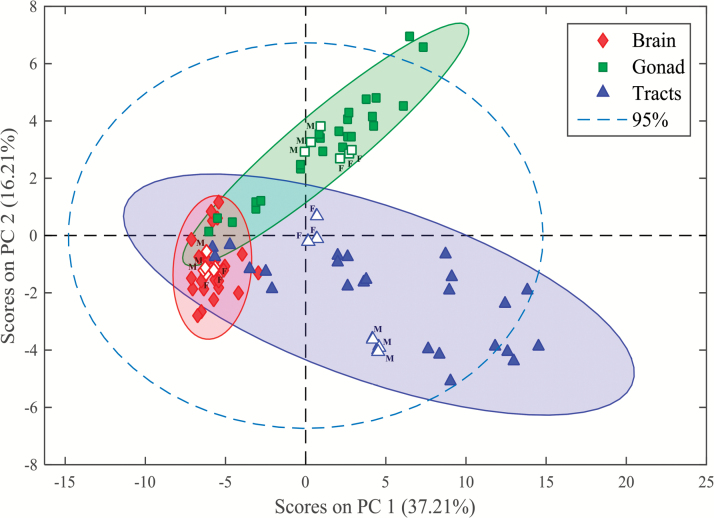
PCA scores plot of the 90 samples analysed (MCR-ALS relative areas of the 94 identified lipid species), corresponding to brain, gonad and gastrointestinal tract tissues of male and female zebrafish including control (open symbols) and stressed-samples (solid symbols). (F: female, M: male).

Each UHPLC-HRMS chromatographic run recorded for every sample resulted in a data file, which was converted to NetCDF format and further imported into MATLAB environment (30). Then, data were loaded into MATLAB workspace and transformed to data matrices, while reducing their size using in-house written routines that search for the regions of interest (ROI) and construct compressed data matrices containing relevant LC-HRMS features (23) without any loss of spectral mass accuracy (see supplementary Figure 1, available at *Mutagenesis* Online). ROI data matrices contain mass spectra at all retention times in their rows and at the ROI selected *m*/*z* values in their columns. In order to compare the distinct samples (control and stressed), new column-wise augmented data matrices were generated, arranging samples one at the top of each other (sample × elution time, *m*/*z* values) (see supplementary Figure 2, available at *Mutagenesis* Online). The final augmented data matrix containing information of the 90 samples was then generated (see supplementary Figure 3, available at *Mutagenesis* Online) in this way. Then, the MCR-ALS method (16) was applied to the final augmented data matrix for the resolution of co-eluted elution profiles and hidden peaks of the different sample constituents (lipids) as well as their corresponding mass spectra. In this analysis, 150 components were simultaneously resolved, each one describing a pure elution and mass spectral profile, and a 99.2% of the total variance was explained (see supplementary Figure 4, available at *Mutagenesis* Online). Also, relative areas of the resolved elution profiles for each of these components in the different analysed samples (control and treatment for the different tissues) were obtained. The elution profiles of the 150 components were further examined and only those that described reliable chromatographic peak features were selected, whereas components explaining background instrumental noise or solvent contribution were excluded. From the spectra profiles of the relevant MCR-ALS components, 94 lipids were finally identified, and from their relative areas, it was possible to evaluate their relative amounts in each sample. A first exploratory evaluation of the calculated MCR-ALS relative areas using a PCA of the 90 samples and 94 variables (lipid species) was performed. Six components were selected to build the model, explaining a cumulative variance of 78.02% (Figure 2). For a detailed examination, and in order to explore potential biomarkers (i.e. components showing significant differences in control *versus* treatment samples), two complementary methods were used. First, a PLS-DA was performed in the data matrix containing calculated areas of the 94 identified lipid species for the 90 samples. PLS-DA was applied 24 times, considering one pair of classes each time (i.e. 4 treatments × 3 tissues × 2 genders). The determination of lipids showing significant differences was feasible when observing variables importance in projection (VIP) scores with a threshold higher than one (see supplementary Figure 5, available at *Mutagenesis* Online). Further, two-sample Student’s *t*-test (*P* < 0.05) was used to corroborate the previously encountered potential biomarkers. Finally, only those lipid species showing significant alterations in both approaches (VIPs from PLS-DA and Student’s *t*-test) were proposed as potential biomarkers (see supplementary Table 1, available at *Mutagenesis* Online). Changes in lipid areas among the distinct sample groups were also examined by performing a hierarchical cluster analysis (HCA) together with headmapping display (31), which also allowed the evaluation of lipid species showing similar behaviour amongst CBNs exposure.

### Peak assignment and identification of lipids

For the identification of the MCR-ALS-resolved components, both a home-made database of lipids built previously (17,32) using the same chromatographic conditions and external databases available online such as LipidMaps (http://www.lipidmaps.org) and Human Metabolome Database were used. The assigned compound corresponded to the lipid molecule with the minimum mass error value (≤10 ppm) respect to the measured *m*/*z*, considering the possible adducts in positive ionization mode. The lipid annotated also had to fulfil an adequate retention time regarding its polarity. Glycerophospholipids, TAG and DAG species were annotated as <lipid subclass> <total fatty acyl chain length> <total number of unsaturated bonds>. In this study, 30 PC, 3 Plasmalogen PC, 3 Lyso PC, 1 Lyso plasmalogen PC, 3 PE, 10 PS, 40 TAG and 4 DAG lipid species were identified (see supplementary Table 2, available at *Mutagenesis* Online).

### Software for LC-MS data analysis

MATLAB 8.6.0 R2015a and R2015b (The MathWorks, Inc., Natick, MA, USA) were used as the development platforms for LC-MS data analysis and visualization. A graphical interface was used to apply MCR-ALS, which additionally provided detailed information about the implementation of this algorithm. Statistics Toolbox™ for MATLAB, PLS Toolbox 7.3.1 (Eigenvector Research Inc., Wenatchee, WA, USA), Bioinformatics Toolbox™ and other home-made routines (30) were used in this work. Waters/Micromass MassLynx™ V 4.1 software was used for data set conversion from raw into NetCDF format and as one of the formula identification platforms through its elemental composition tool.

### Surface-enhanced Raman spectroscopy

SERS was performed using nanosilver, supplied from Dr Cui Li’s Lab (33,34). Three spectra were acquired from each sample (three samples for each group). Silver nanoparticles (Ag NPs) for SERS were synthesised using trisodium citrate as the reductants (33,34). For SERS interrogation, the lipid extracts in MTBE were mixed with Ag NPs. After vortexing, an aliquot of 10 µL of the mixture were dropped onto a glass slide for SERS measurement.

SERS spectra were acquired using a LabRAM Aramis (HORIBA JobinYvon) confocal micro-Raman system equipped with a 1200 g mm^−1^ grating, helium–neon 632.8 nm laser (laser power ≤70 mW prior to lens). System calibration was carried out using a silicon calibration source for wavenumber shift. A 50× objective (Olympus) with a numerical aperture of 0.55 was used to focus the laser beam and collect Raman signal with a working distance of about 8 mm. In order to reduce any laser damage to cells, DuoScan in the micromapping mode with a scanning area of 30 µm × 30 µm was applied with an acquisition time of 5 s.

Spectral data processing, acquired from Raman spectroscopy, were performed using IRootLab toolbox (http://irootlab.googlecode.com) running on MATLAB r2010a (The MathWorks, Inc., USA) (35). SERS spectra were initially processed in LabSpec 6 (software provided with the Raman system) for cosmic ray removal and background correction. The spectral data were then inputted into MATLAB and preprocessed following wavelet de-noising, cut to 400–1800 cm^−1^, second differentiation and vector normalisation (36). The differentiation is employed both as a means of baseline correction and to resolve overlapped bands (23). The wavelet de-noising utilises non-linear filtering implemented through multi-scale decomposition and thresholding for de-noising, especially for the spectra containing sharp peaks. Computational analysis using multivariate techniques including PCA and linear discriminant analysis (LDA) can efficiently analyse such large spectral data sets (24,37). The main difference between PCA and LDA is that the former is an unsupervised method, whilst the latter is supervised. PCA looks for projections to maximise variance and LDA looks for projections that maximise the ratio of between-category to within-category scatter. Following preprocessing, PCA was applied to the data set. PCA reduces the dimensions of the data. Undoubtedly, PCA is capable of identifying important information in spectral data but has less discriminatory power. Often, in order to interpret complex biochemical information with labelled classes, further analysis using supervised procedures like LDA is required (37). The output data derived from PCA or PCA-LDA (the first 10 PC factors of PCA are used for LDA since >99% of variance is captured) can then be visualised as 1-D, 2-D or 3-D scatterplots (‘scores plots’). In scores plots, nearness between two categories implies similarity, while distance indicates dissimilarity. To reveal the biochemical alterations associated with each category in the data set, both loading plots and cluster vectors were developed. To simplify the identification of the main biochemical alterations distinguishing each category, peak detector was used to indicate the first few highest peaks in the loadings plots or cluster vectors plots.

### Global DNA methylation in brain tissue determined by HPLC-MS

Brain tissue in zebrafish exposed to one of four CBNs at 0.1 mg L^−1^ were collected and stored in liquid nitrogen prior to DNA methylation analysis. DNA was extracted from tissue using the DNeasy tissue Kit (Tiangen, China) following the manufacturer’s instructions. RNase A was added to columns in the kit to remove RNA residue. DNA hydrolysis was conducted using a mixture degradase kit (DNA Degradase Plus, Zymo Research, USA) following the manufacturer’s protocol. To confirm complete hydrolysis of DNA, agarose gel electrophoresis was employed to test the result of the hydrolysis. The DNA hydrolysis mixtures were then stored at −20°C for mass spectrometric analysis.

To perform chromatographic separation, a Kinetex C_18_ column (100 mm × 4.6 mm, 2.6 µm; Phenomenex, USA) was employed in an HPLC system (LC-20AD, Shimadzu, Japan). The injection volume was 20 µL. The mobile phase consisted of water (A) and methanol (B). A gradient elution protocol was as follows: 0–0.01 min, 3% B; 0.01–5.00 min, 5% B; 3.00–12.00 min, 50% B; 12.00–15.00 min, 100% B; 15.00–25.00 min, 3% B at a flow rate of 0.5 mL min^−1^. For the mass spectrometric analysis, an electrospray ionization tandem mass spectrometry (LCMS-8030, Shimadzu, Japan) system was used, operating in positive ionization mode and conditioned at a capillary temperature of 400°C and medium nitrogen curtain gas. Optimised multiple reaction monitoring conditions were set to evaluate dC from *m*/*z* 228.1 to 111.9 and 5 mdC from *m*/*z* 242.1 to 126.0. Data acquisition and processing were performed *via* Analyst software. The global DNA methylation ratio (MR) was determined by MR = [5 mdC]/([5 mdC] + [dC]).

The data are expressed as the mean ± SD. Significant differences among multiple groups were determined using a one-way analysis of variance (ANOVA) followed by Dunnett’s *post hoc* tests. Probabilities of *P* < 0.05 were considered as statistically significant. All these tests were conducted in GraphPad Prism 4 (GraphPad Software, USA).

## Results

Post-exposure to CBNs, tissues (brain, gonads and gastrointestinal tracts) were isolated from zebrafish (male and female). Isolation of lipids allowed for lipidomic analysis. PCA of the 90 samples (MCR-ALS relative lipid areas) are shown in Figure 2. The resultant scores plot indicates that effects produced are more pronounced on lipid extracts from gonads and intestinal tracts compared to brain. This is reflected by the higher data dispersion observed in the first two clusters (gonads and intestinal tracts) compared with the latter (i.e. brain).

**Figure 3.**
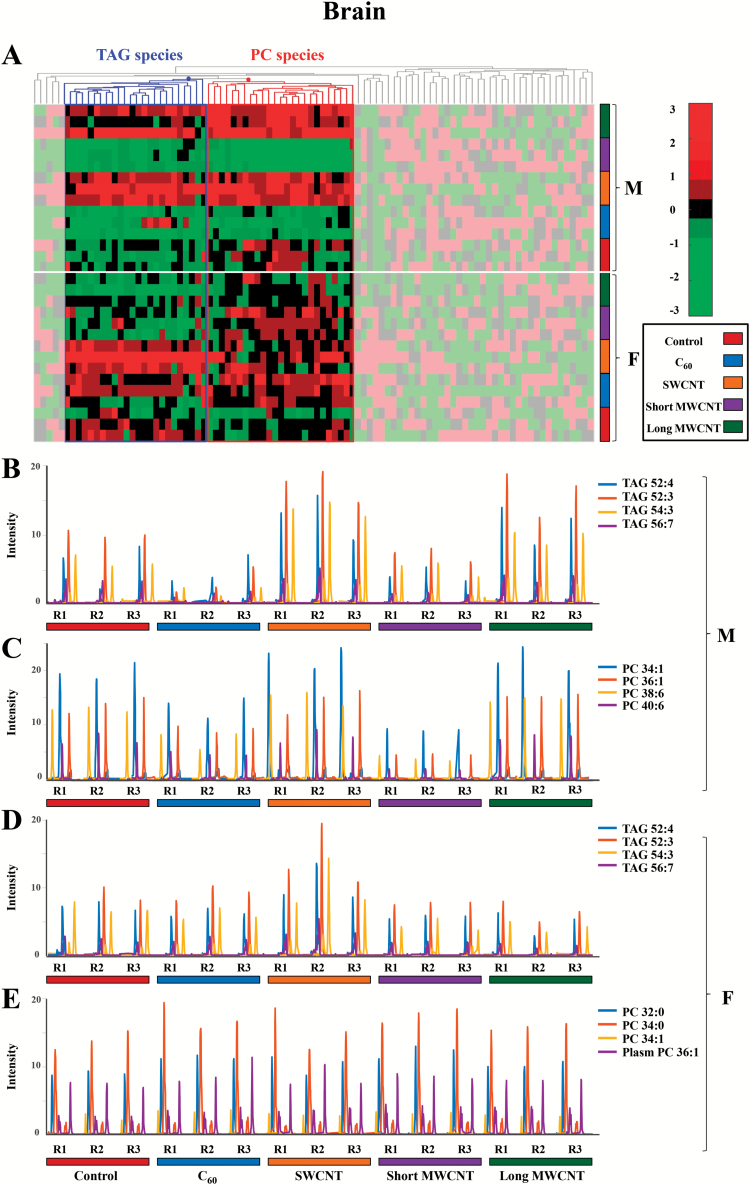
(**A**) Hierarchical clustering heatmap of the peak areas of the 94 identified lipid species in brain zebrafish samples (autoscaled data). Degree of change in the different stressed groups (C_60_, SWCNT, short MWCNT and long MWCNT) compared with the control groups is marked with colours inside the heatmap indicating up-regulation (red) and down-regulation (green), as indicated by the colour bar at the top right of figure. Identified lipids are represented in the horizontal axis and sample groups in the vertical axis. Two clusters corresponding to glycerolipids (mainly TAG species) and glycerophospholipids (mainly PC species) are outlined. The decoloured parts of the heatmap correspond to lipid species showing no clear disrupting tendency. (**B** and **C**) Elution profiles of some representative TAG and PC lipid species, respectively, from male brain tissue samples exposed to CBNs. (**D** and **E**) Elution profiles of some representative TAG and PC lipid species, respectively, from female brain tissue samples exposed to CBNs. (F: female, M: male, R: replicate, 1 to 3: replicate number). The following colour legend for the distinct treatments is used: controls (deep red), C_60_ (blue), SWCNT (orange), short MWCNT (purple) and long MWCNT (dark green).

It remains important to determine whether dietary exposure to CBNs results in exposure to distal and protected tissues such as brain. Figure 3 shows that in males, TAG and PC species behave similarly, showing a decrease in their intensities compared with the controls in C_60_- and short MWCNT-exposed tissue samples (blue and violet, respectively), whereas there is an increase in these lipid species in SWCNT- and long MWCNT-exposed tissue samples (orange and dark green, respectively). However, no obvious effects in PC species are noted in CBN-exposed females. TAG species appear to be elevated in SWCNT-exposed samples (orange), but there is a slight decrease in their levels in short MWCNT- and long MWCNT-exposed samples (violet and dark green, respectively).

**Figure 4.**
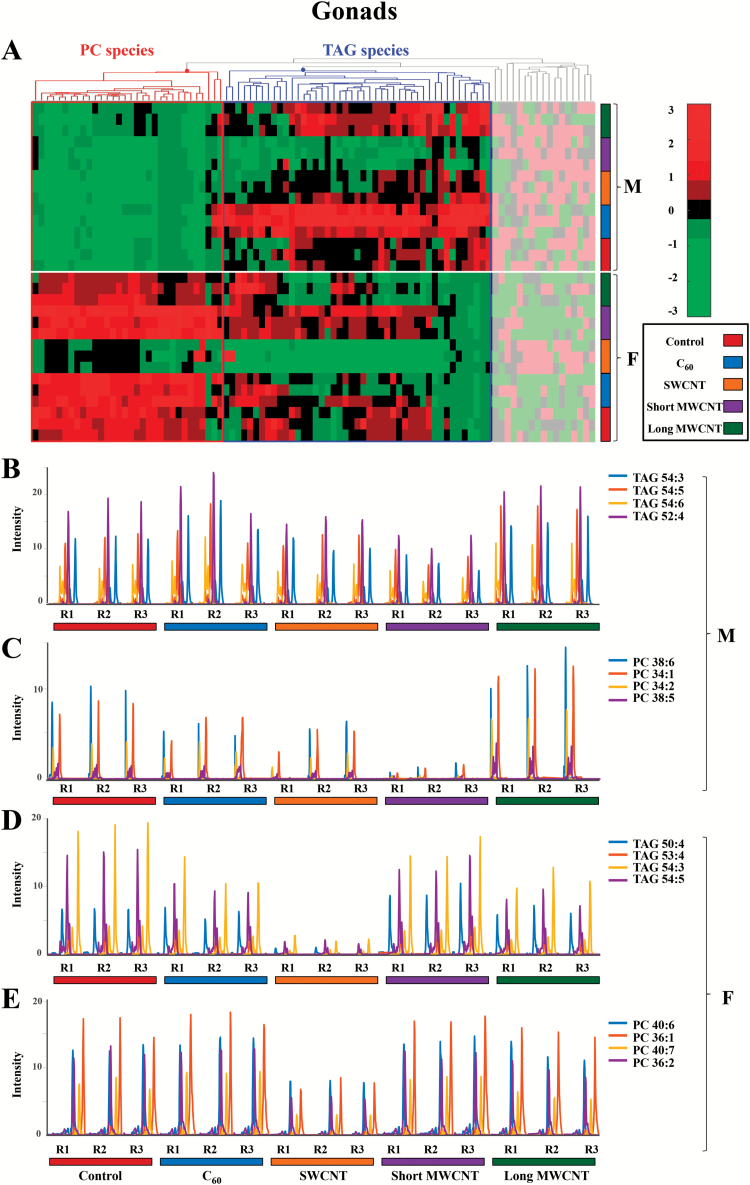
(**A**) Hierarchical clustering heatmap of the peak areas of the 94 identified lipid species in gonad zebrafish samples (autoscaled data). Degree of change in the different stressed groups (C_60_, SWCNT, short MWCNT and long MWCNT) compared with the control groups is marked with colours indicating up-regulation (red) and down-regulation (green), as indicated by the colour bar at the top right of figure. Identified lipids are represented in the horizontal axis and sample groups in the vertical axis. Two clusters corresponding to glycerolipids (mainly TAG species) and glycerophospholipids (mainly PC species) are outlined. The decoloured parts of the heatmap correspond to lipid species showing no clear disrupting tendency. (**B** and **C**) Elution profiles of some representative TAG and PC lipid species, respectively, from male gonad tissue samples exposed to CBNs. (**D** and **E**) Elution profiles of some representative TAG and PC lipid species, respectively, from female gonad tissue samples exposed to CBNs. (F: female, M: male, R: replicate, 1 to 3: replicate number). The following colour legend for the distinct treatments is used: controls (deep red), C_60_ (blue), SWCNT (orange), short-MWCNT (purple) and long MWCNT (dark green).

A major concern regarding CBN exposure would be with regard to the effects on reproductive health. In zebrafish, there appears to be male *versus* female differences in that PC and TAG species behave differently in males, whereas in females they exhibit a similar pattern (Figure 4). In males, TAG species appear to decrease in short MWCNT-exposed tissue samples (violet), but there is an apparent increase in C_60_- and long MWCNT-exposed tissue samples (blue and dark green, respectively). PC species exhibit a marked increase in long MWCNT-exposed samples (dark green). In females, both TAG and PC lipids exhibit remarkable decreases in levels compared with the controls in SWCNT-exposed tissue samples (orange).

**Figure 5.**
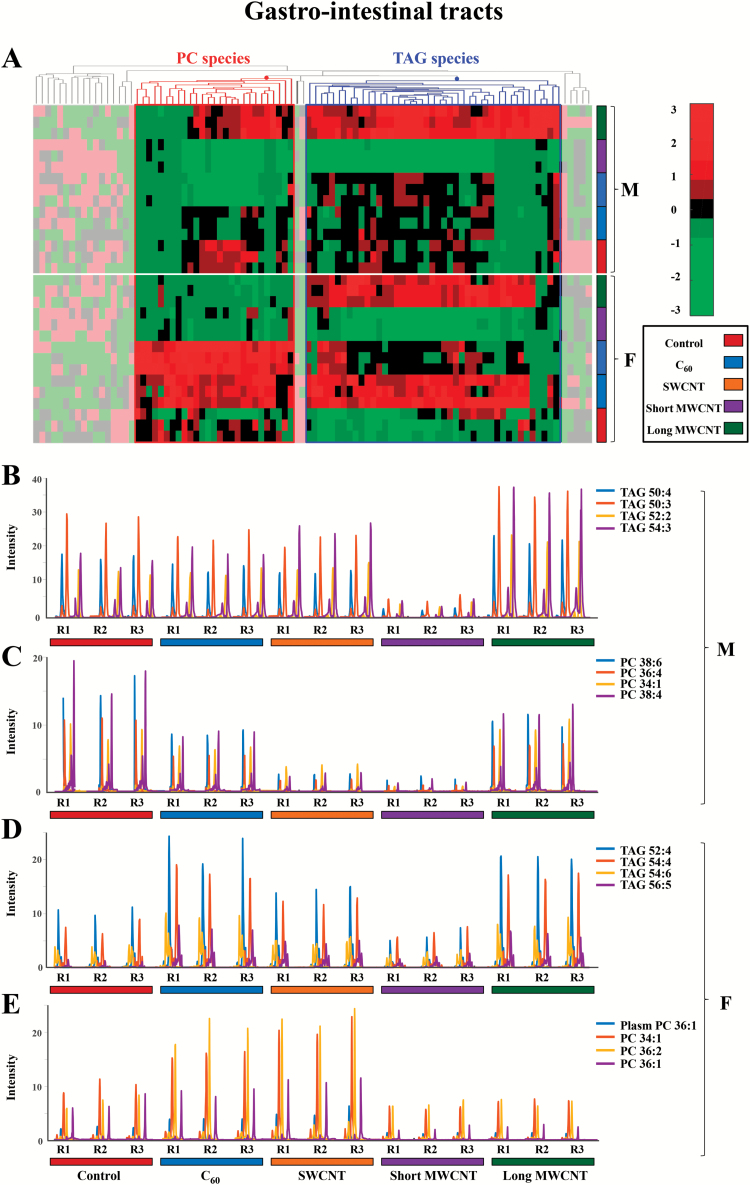
(**A**) Hierarchical clustering heatmap of the peak areas of the 94 identified lipid species in gastrointestinal tract zebrafish samples (autoscaled data). Degree of change in the different stressed groups (C_60_, SWCNT, short MWCNT and long MWCNT) compared with the control groups is marked with colours inside the heatmap indicating up-regulation (red) and down-regulation (green), as indicated by the colour bar at the top right of figure. Identified lipids are represented in the horizontal axis, and sample groups in the vertical axis. Two clusters corresponding to glycerolipids (mainly TAG species) and glycerophospholipids (mainly PC species) are outlined. The decoloured parts of the heatmap correspond to lipid species showing no clear disrupting tendency. (**B** and **C**) Elution profiles of some representative TAG and PC lipid species, respectively, from male gastrointestinal tract tissue samples exposed to CBNs. **(D** and **E**) Elution profiles of some representative TAG and PC lipid species, respectively, from female gastrointestinal tract tissue samples exposed to CBNs. (F: female, M: male, R: replicate, 1 to 3: replicate number). The following colour legend for the distinct treatments is used: controls (deep red), C_60_ (blue), SWCNT (orange), short-MWCNT (purple) and long MWCNT (dark green).

As a tissue that would readily come into contact with CBNs *via* dietary exposure, effects on lipid constituents in gastrointestinal tracts from male and female zebrafish were examined (Figure 5). In males, TAG species show decreases due to short MWCNT treatment (violet), whereas there are increases due to long MWCNT exposure (dark green). PC species exhibit a decrease caused by SWCNT or short MWCNT exposures (blue and dark green, respectively). In females, TAG species increase after C_60_ or long MWCNT exposure (blue and dark green, respectively), but appear decreased following short MWCNT treatment (violet). PC species are elevated after C_60_ or SWCNT exposures (pink and blue, respectively) and decreased post-exposure with short MWCNT or long MWCNT (violet and dark green, respectively).

**Figure 6.**
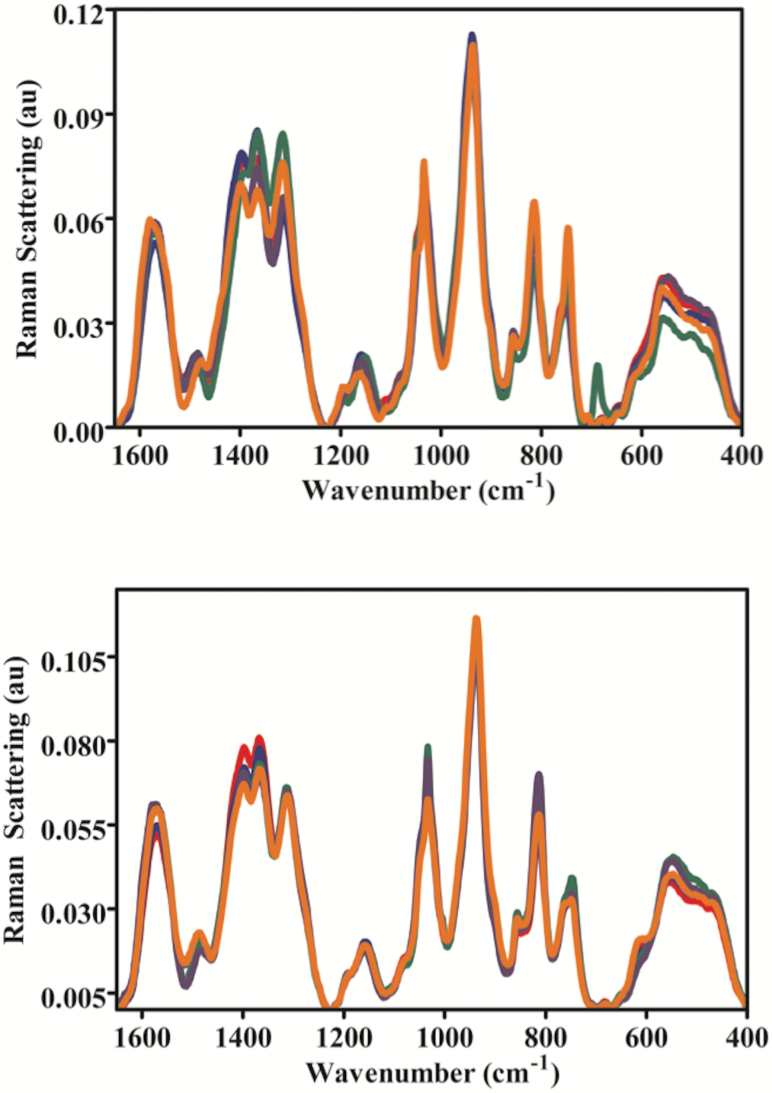
SERS spectra of lipid extracts of brain tissue from male (upper panel) and female (lower panel) zebrafish post-exposure with one of four CBNs. Fish (2 males and 2 females) exposures were conducted in 10 L glass tanks. CNP exposure (0.1 mg L^−1^) was initiated and run for 21 days. Post-exposure and isolation of brain tissue, lipids were extracted; these were then mixed with Ag NPs prior to SERS. The spectral data were then inputted into MATLAB and preprocessed following wavelet de-noising, cut to 400–1800 cm^−1^, second differentiation and vector normalisation. —, Control;—, C_60_ fullerene-treated; —, Long MWCNT-treated; —, Short MWCNT-treated; and, —, SWCNT-treated.

**Figure 7.**
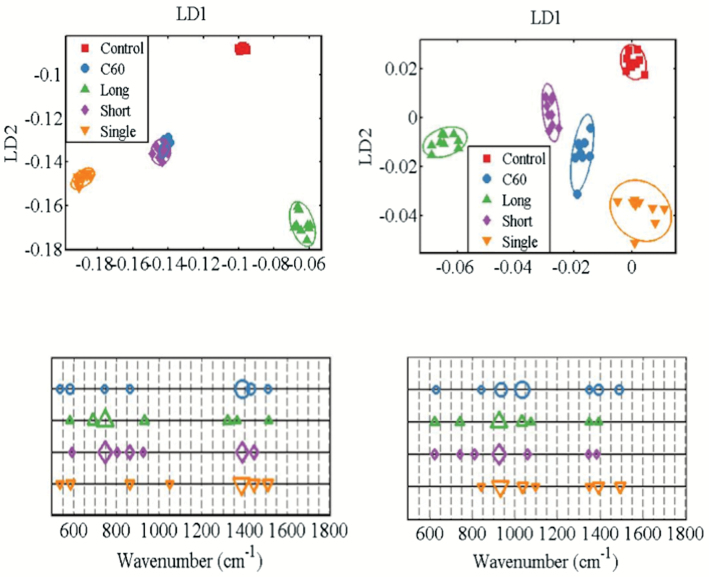
Scores plots and corresponding cluster vectors plots of brain tissue from male (left panels) and female (right panels). Fish (2 males and 2 females) exposures were conducted in 10 L glass tanks. CNP exposure (0.1 mg L^−1^) was initiated and run for 21 days. Post-exposure and isolation of brain tissue, lipids were extracted; these were then mixed with Ag NPs prior to SERS. Following spectral preprocessing, computational analysis using the multivariate techniques of PCA and LDA were applied. Score plots show clusters for control (red squares), C_60_ fullerene-treated (blue diamond), long MWCNT-treated (green triangle), short MWCNT-treated (purple diamond) and SWCNT (inverted orange triangle). Cluster vectors plots indicate the top distinguishing wavenumbers associated with each CBN treatment compared with the control; symbol size is proportional to strength of alteration.

**Figure 8.**
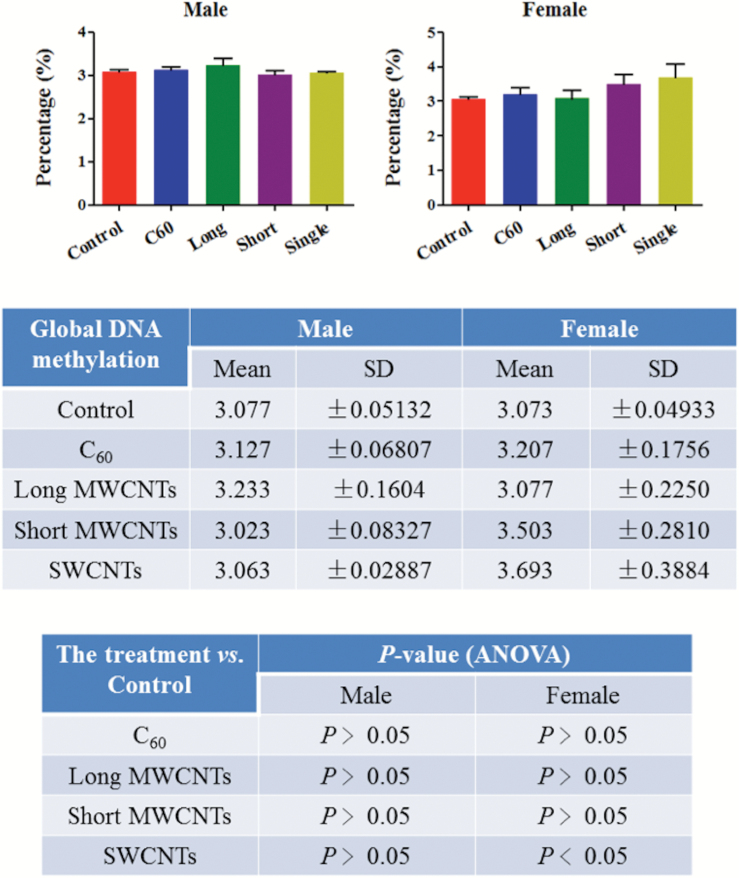
Levels of genomic methylation in brain tissue of male or female zebrafish post-exposure with one of four CBNs. Fish (2 males and 2 females) exposures were conducted in 10 L glass tanks. CNP exposure (0.1 mg L^−1^) was initiated and run for 21 days. Post-exposure and isolation of brain tissue, DNA methylation analysis was carried out.

To further examine CBN-induced effects in brain tissues of zebrafish, a SERS approach was employed (Figure 6). The preprocessed SERS spectra show that CBNs induce lipid alterations in both males and females, but there are obvious gender-specific differences. In both males and females, long MWCNTs appear to induce the most profound effects followed by SWCNTs as further evidenced by their cluster separation away from control in the resultant PCA-LDA scores plot (Figure 7A). Whilst clusters for C_60_ and short MWCNTs were still significantly removed away from the control cluster, they appear to co-locate and thus more likely to generate similar alterations. The cluster vector plots (Figure 7B) supported the notion that CBNs individually induced nanoparticle-specific profiles of alterations in males, whereas altered features in lipid extracts derived from female zebrafish brains were more similar. Table 1 shows the tentative wavenumber assignments associated with dietary CBN exposure; in females, there are consistent similarities although SWCNT-exposed differs from the other three treatments. This consistency is not mirrored in the highlighted tentative wavenumber alterations in CBN-exposed male zebrafish. In addition to the lipid alterations noted, there also appears to be consistent elevations in global genomic methylation; this was most profound in female zebrafish brain tissue samples following exposure to short MWCNTs and SWCNTs (*P* < 0.05; Figure 8).

**Table 1.** Primary wavenumbers (ranked in order of importance) in cluster vectors derived from PCA-LDA of SERS spectral data sets.

	Female		Male	
CBN	Wavenumber (cm^−1^)	Tentative assignments	Wavenumber (cm^−1^)	Tentative assignments
C_60_ fullerene	1033	Phenylalanine	1387	CH_3_
	932	C–C stretch	1430	CH_2_ bending
	1393	CH_3_	582	C–C twisting mode
	1488	CN stretch	743	C–S stretch
	838	O–P–O stretch	862	O–P–O stretch
	1346	CH	533	ν(S–S)
	626	C–C twisting mode	1510	ν(C=C)
Long MWCNTs	924	C–C stretch	745	C–S stretch
	1033	Phenylalanine	689	C–C twisting mode
	741	C–S stretch	930	C–C stretch
	621	C–C twisting mode	1321	CH
	1072	Chain C–C stretch	1364	CH_3_
	1393	CH_3_	1512	ν(C=C)
	1346	CH	580	ν(S–S)
Short MWCNTs	924	C–C stretch	745	C–S stretch
	1058	Chain C–C stretch	1387	CH_3_
	807	O–P–O stretch	860	O–P–O stretch
	621	C–C twisting mode	1445	CH_2_ bending
	741	C–S stretch	589	ν(S–S)
	1383	CH_3_	924	C–C stretch
	1344	CH	803	O–P–O stretch
SWCNTs	930	C–C stretch	1385	CH_3_
	1393	CH_3_	1443	CH_2_ bending
	1035	Phenylalanine	1510	ν(C=C)
	1494	CN stretch	862	O–P–O stretch
	838	O–P–O stretch	584	ν(S–S)
	1095	ν(C–N)	533	ν(S–S)
	1346	CH	1049	Phenylalanine

## Discussion

Given the rising ubiquity of CBN environmental exposure, there is now an urgent need to assess risk under relevant conditions. Herein, we exposed male and female zebrafish to CBNs *via* their diet. Levels employed would be consistent with reasonable low environmental levels (38). Although mechanically disaggregated, CBN solutions would reasonably be expected to be stable, especially CNT solutions (29,39), although C_60_ would be more likely to agglomerate (40–42). We then examined the lipidomic profile of exposed *vs.* control in brain, gonads and gastrointestinal tissues. Whilst the liver acts as a portal to the body in many sentinel organisms (43), only in a small number of cases is it the target for consequent pathology. The tissues chosen in this study were chosen to determine whether CBNs possess potential to induce developmental alterations (i.e. brain) reproductive alterations (i.e. gonads) or point-of-contact alterations (i.e. gastrointestinal tract). From an exploratory study, marked alterations were noted (Figures 2 to 5). To confirm brain-associated alterations, an SERS approach was applied to the same lipid extracts and significant spectral alterations (Figures 6 and 7A) were noted associated with tentative chemical bonds (Figure 7B, Table 1). Interestingly, the observed lipidomic alterations in brain tissues using both approaches (i.e., LC-HRMS and SERS) were to some extent correlated. As evidenced in the scores plots obtained following SERS analysis (Figure 7A), treatments with long MWCNTs and SWCNTs produced most pronounced effects in both male and female brain tissues, whereas C_60_ and short MWCNTs treatments produced minor and similar alterations (co-located clusters in Figure 7A).

Accordingly, LC-MS lipid profiles of male brain tissues evidenced a large impact of long MWCNTs and SWCNTs, both producing an increment in TAG and PC lipid species, while there is a minor impact of C_60_ and short MWCNTs treatments, each behaving similarly and producing a diminution on TAG and PC lipid levels. Moreover, both SERS and LC-MS approaches evidenced that SWCNT treatment caused major alterations in female brain tissues, showing a discriminant pattern in comparison with the other treatments. Such distinguished effects caused by SWCNTs are noticeable in the cluster vectors plots resulting from the SERS approach (Figure 7B) but become even more evident in LC-MS TAG profiles of brain female samples represented in Figure 3D. In the latter Figure, it is readily observed that SWCNT treatment produces a significant increment in TAG species, while the other CBN treatments produce no significant alterations respect to controls.

Sparse evidence is found in the literature regarding the effects that CBNs can pose on lipid molecules of organisms. Little research in this direction has focused on the study of the interaction between CBNs and membrane lipids, such as PC species and their derivatives with plasmalogens. Rusciano *et al.* (44) investigated the interaction of organic carbon-based nanoparticles (NOC) with giant unilamellar vesicles, used as a model membrane, and suggested that ROS production induced by NOCs causes membrane peroxidation leading to the alteration of membrane permeability. Moreover, Engelmann *et al.* (45) suggested a direct linkage between the induced alterations on membrane lipids and the generation of ROS. Such connection is attributed to the reported capacity of plasmenyl-PCs to protect against the damaging effects of ROS, facilitating signalling processes and protecting membrane lipids from oxidation. In this study, a significant increase of PC species and derivatives was observed on brain male samples exposed to SWCNTs or long MWCNTs, on gonad male samples exposed to long MWCNTs and on gastrointestinal female samples treated with C_60_ or SWCNTs. The increment of the amount of such membrane lipids can be attributed to a defence mechanism of the cellular membranes against CBN-induced oxidative stress. Concerning the effects that CBNs may have on lipid droplets, mainly formed by TAG and DAG species, no evidence currently exists in the literature. However, in this study, we have demonstrated the capacity of CBNs to alter TAG and DAG species in brain, gonad and gastrointestinal tissue samples of zebrafish. Further work would require extensive transmission electron microscopy studies to determine uptake and localisation of tissue-specific CBNs (46).

Finally, we examined whether CBN exposure might alter the levels of genomic methylation; despite expected *in vivo* heterogeneity, a consistent increase following CBN exposure is noted especially post-exposure with SWCNTs (Figure 8). Given the likely persistence of these agents, long-term exposure would likely lead to modifications that could alter development.

Overall, this work provides insights on the effects that CNTs or fullerenes can pose on lipids of female and male zebrafish (*Danio rerio*) at low environmentally relevant doses and highlights: (i) the ability of CBNs to induce marked lipid alterations in brain, gonads and gastrointestinal tissue samples of zebrafish; (ii) the response of membrane lipids against the oxidation damage induced by CBNs; and (iii) the capacity of CBNs to cause elevations in global genomic methylation, especially in female zebrafish tissue samples. Hence, this study proves that dietary exposures to low environmentally relevant levels of CBNs can induce far-reaching alterations in a range of tissues. These would likely be sublethal but could modify consequent susceptibility to other influences such as environmental exposures (47) and disease onset. There will be an increasing need for sophisticated data-handling approaches to tease out exposure-specific effect alterations in target cells following the application of omic approaches (47). Future studies are needed to explore the long-term consequences of these CBN exposures as well as their influence in combination with other environmental influences such as chemicals.

## Supplementary data

Supplementary Tables 1 and 2 and Supplementary Figures 1 to 5 are available at *Mutagenesis* Online.

## Funding

The research leading to these results has received funding from the European Research Council under the European Union’s Seventh Framework Programme (FP/2007–2013)/ERC grant agreement no. 320737. One of the first authors (E.G.) acknowledges the Spanish Government (Ministerio de Educación, Cultura y Deporte) for a predoctoral FPU scholarship (FPU13/04384). Research in F.L.M.’s laboratory is supported by Rosemere Cancer Foundation and the Engineering and Physical Sciences Research Council (EPSRC; grant no: EP/K023349/1). Data relating to this study is deposited and freely available at: https://dx.doi.org/10.6084/m9.figshare.4012683.v1.

Conflict of interest statement: None declared.

## Supplementary Material

Supplementary Data
