# Supplementary material for: Diet-sourced carbon-based nanoparticles induce lipid alterations in tissues of zebrafish (Danio rerio) with genomic hypermethylation changes in brain
Source: Mutagenesis. 2016 Oct 26;32(1):91–103. doi: 10.1093/mutage/gew050 (PMC5180172; doi:10.1093/mutage/gew050)
Supplement: Supplementary Data [file supp_32_1_91__index.html]

Diet-sourced carbon-based nanoparticles induce lipid alterations in tissues of zebrafish (Danio rerio) with genomic hypermethylation changes in brain — Diet-sourced carbon-based nanoparticles induce lipid alterations in tissues of zebrafish (Danio rerio) with genomic hypermethylation changes in brain — Supplementary Data 

# Diet-sourced carbon-based nanoparticles induce lipid alterations in tissues of zebrafish (*Danio rerio*) with genomic hypermethylation changes in brain

## Supplementary Data

Data files

- Supplementary Data - Supplementary Data
