# Supplementary material for: Diet-sourced carbon-based nanoparticles induce lipid alterations in tissues of zebrafish (Danio rerio) with genomic hypermethylation changes in brain
Source: Mutagenesis. 2016 Oct 26;32(1):91–103. doi: 10.1093/mutage/gew050 (PMC5180172; doi:10.1093/mutage/gew050)
Supplement: Supplementary Data [file supp_gew050_Supplementary_Information_Final.pdf]

Supplementary Information

Diet-sourced carbon-based nanoparticles induce lipid alterations in tissues of zebrafish (*Danio rerio*) with genomic hypermethylation changes in brain

Eva Gorrochategui[1,§], Junyi Li[2,§], Nigel J. Fullwood[3], Guang-Guo Ying[4], Meiping Tian[5], Li Cui[5], Heqing Shen[5], Sílvia Lacorte[1], Romà Tauler[1], Francis L. Martin[2,*]

[1]Department of Environmental Chemistry, Institute of Environmental Assessment and Water Research (IDAEA), Consejo Superior de Investigaciones Científicas (CSIC), Barcelona, 08034, Catalonia, Spain; [2]Centre for Biophotonics, Lancaster Environment Centre, Lancaster University, Lancaster LA1 4YQ, UK; [3]Biomedical and Life Sciences Division, Lancaster Environment Centre, Lancaster University, Lancaster LA1 4YQ, UK; [4]State Key Laboratory of Organic Geochemistry, Guangzhou Institute of Geochemistry, Chinese Academy of Sciences, Guangzhou 510640, China; [5]Key Lab of Urban Environment and Health, Institute of Urban Environment, Chinese Academy of Sciences, Xiamen 361021, China

Figure S1. LC-MS profiles once imported into MATLAB environment and after compression and data matrix construction. Example shown for lipid extracts from control brain, gonads and gastro-intestinal tracts of female zebrafish.

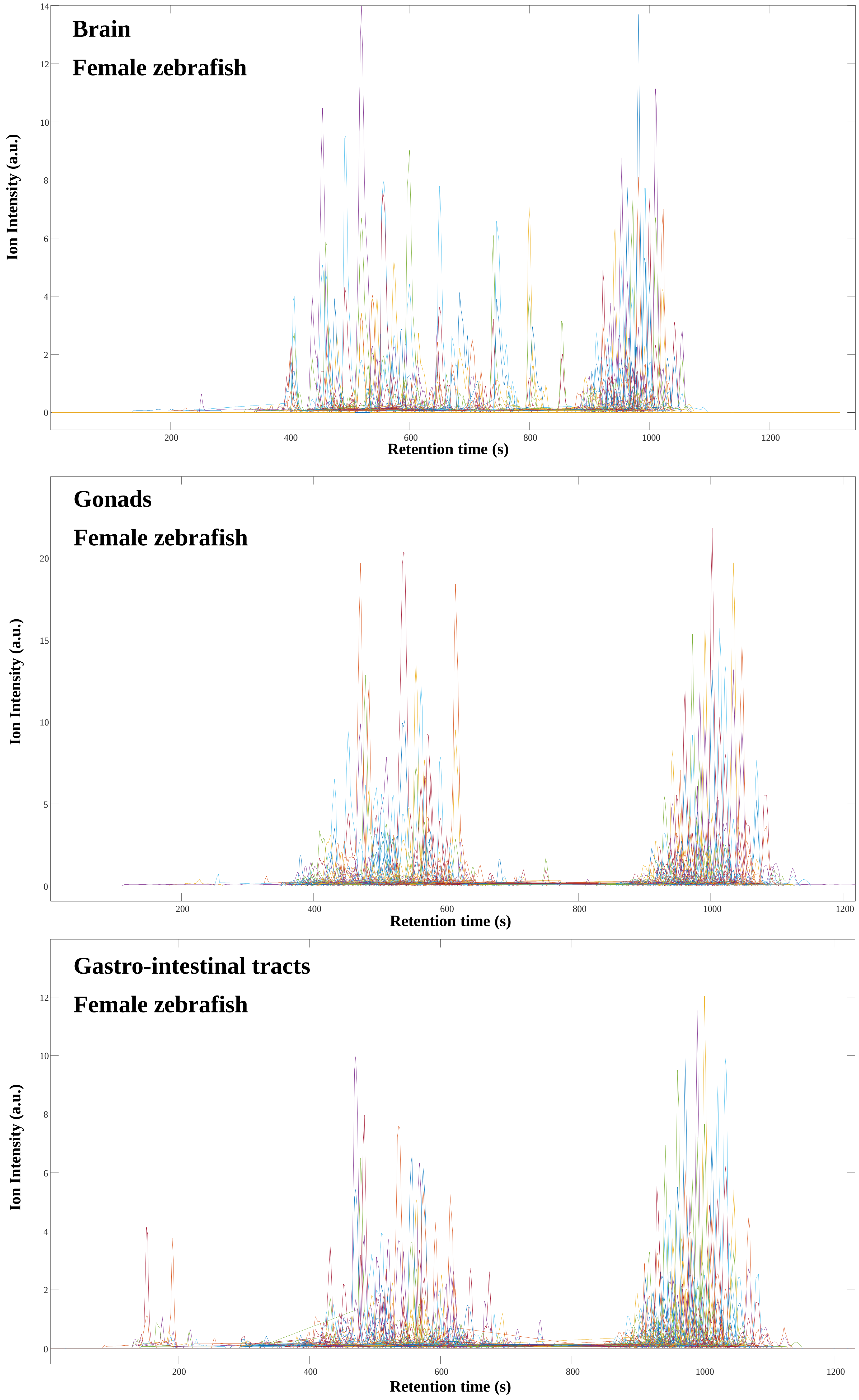

Figure S2. Augmented LC-MS data matrices of brain samples (Control, C_{60}, SWCNT, short MWCNT and long MWCNT) of female and male zebrafish.

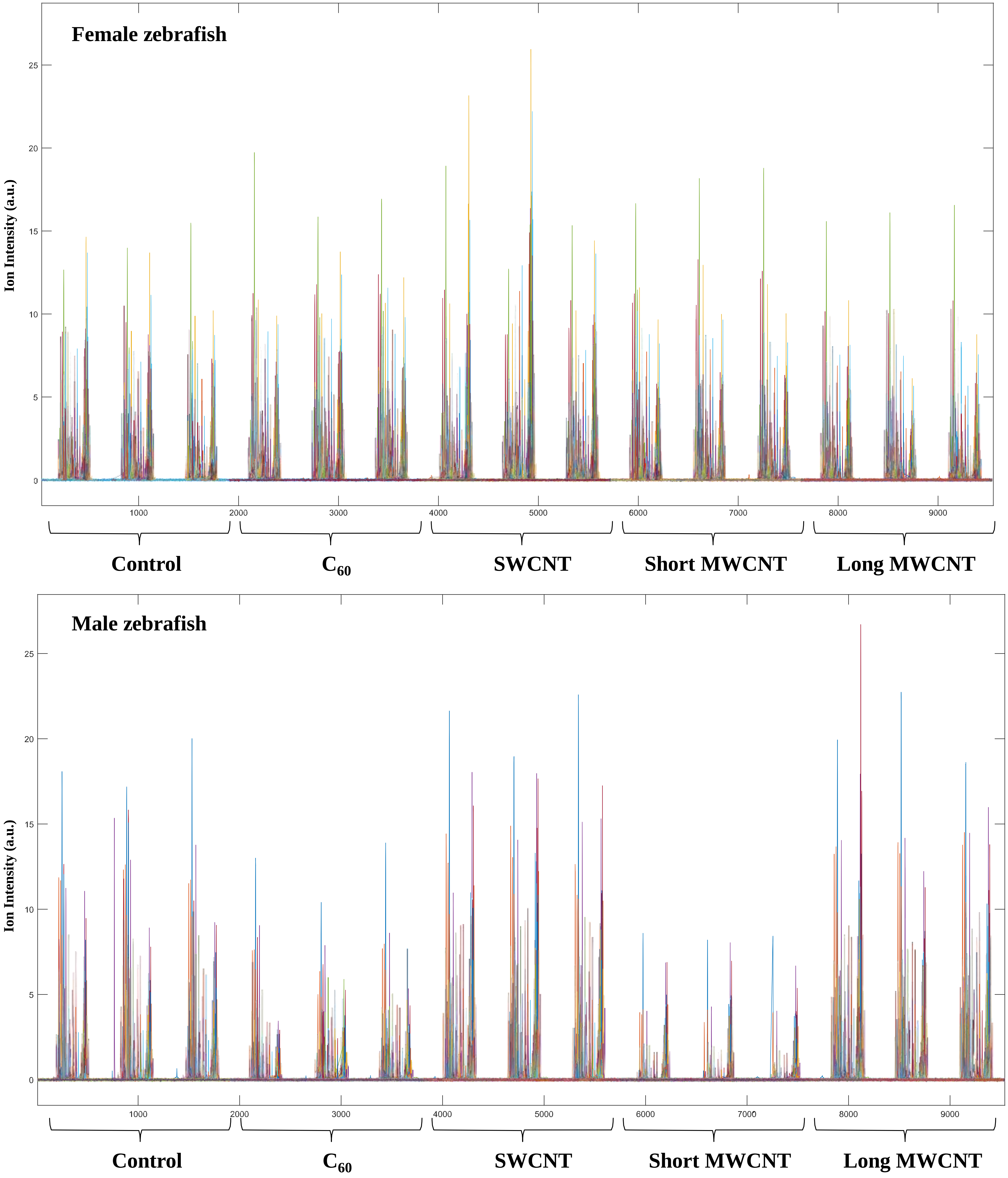

Figure S3. Final augmented LC-MS data matrix containing information of the 90 samples analysed. Input matrix for further MCR-ALS analysis.

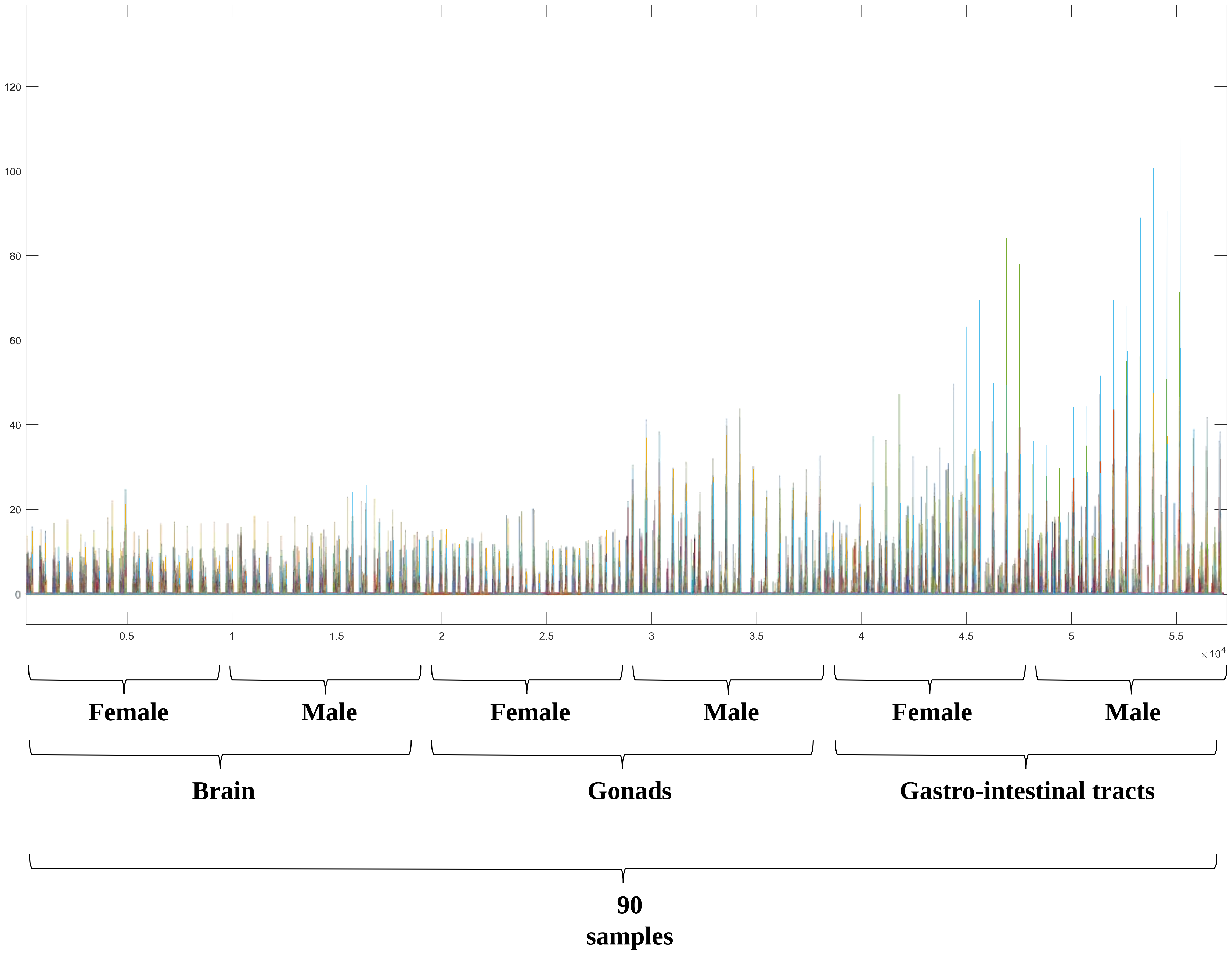

Figure S4. A) Output of MCR-ALS analysis of the final augmented data matrix to find purest elution and mass spectra profiles. 150 components resolved, explaining 99.2% of data variance. **B)** Example of elution and spectra profiles for component 2 in the 90 samples.

A]

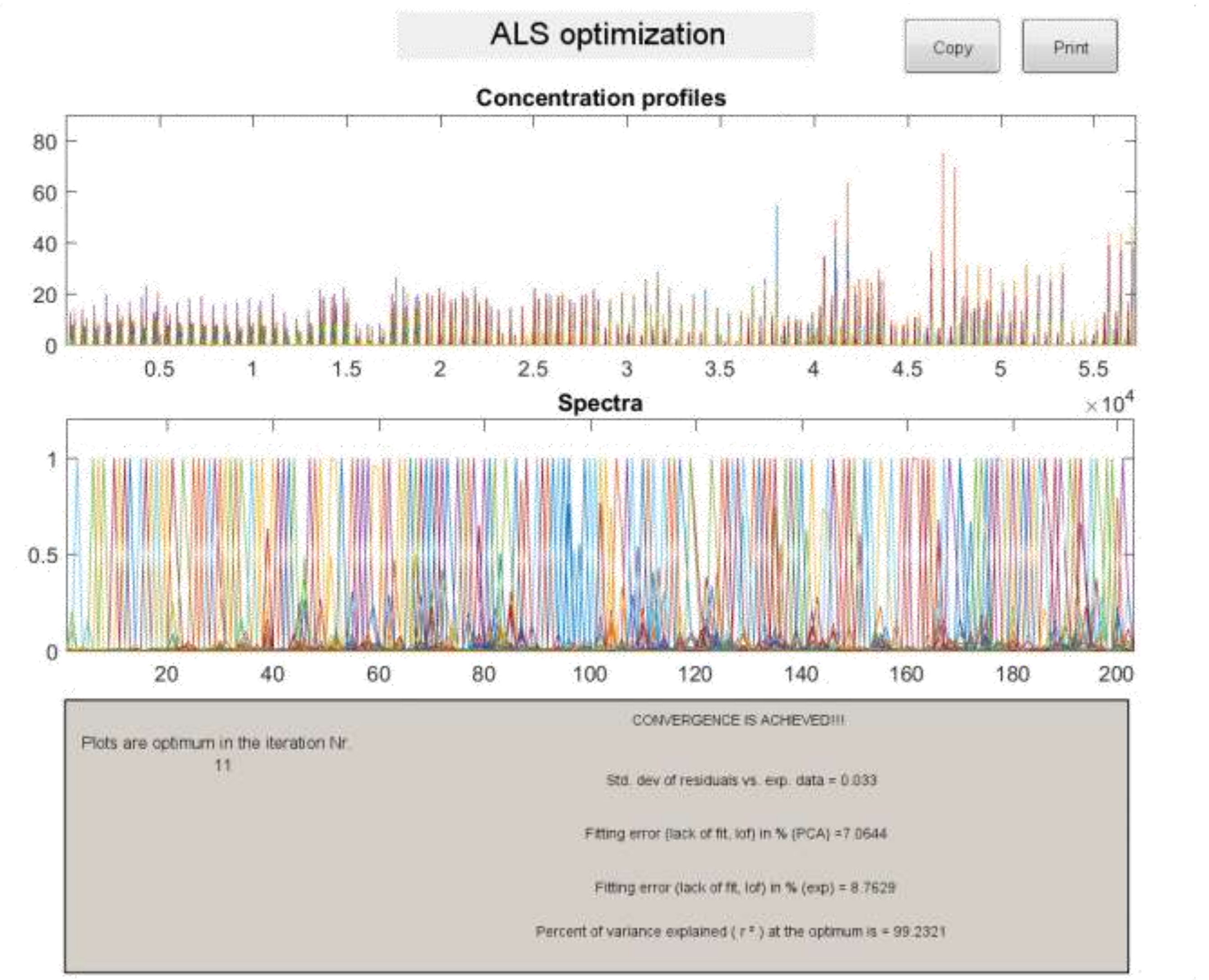

B]

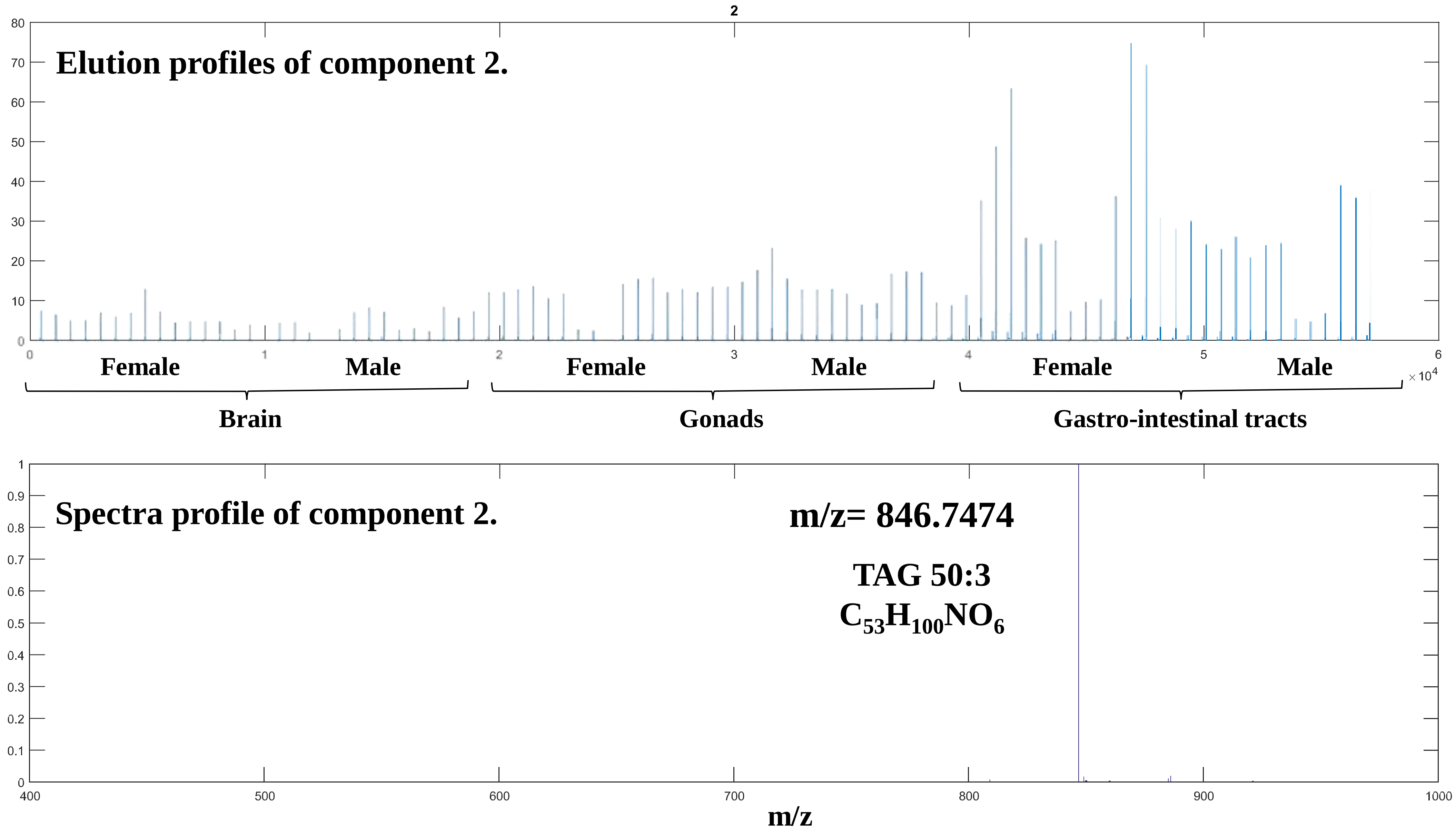

Figure S5. A) PLS-DA scores plot for control and C_{60}-stressed samples of brain tissues of female zebrafish**. B)** Variables importance in projection (VIP scores) plot from PLS-DA analysis of the 94 lipid species. Horitzontal red line show the threshold values of 1 (dotted line) lipid species in red indicate most important variables according to the highest threshold value (potential biomarkers). (* Lipid species also showing significant differences among controls and stressed samples in the two-sample Student's *t*-Test ($P < 0.05$)).

A]

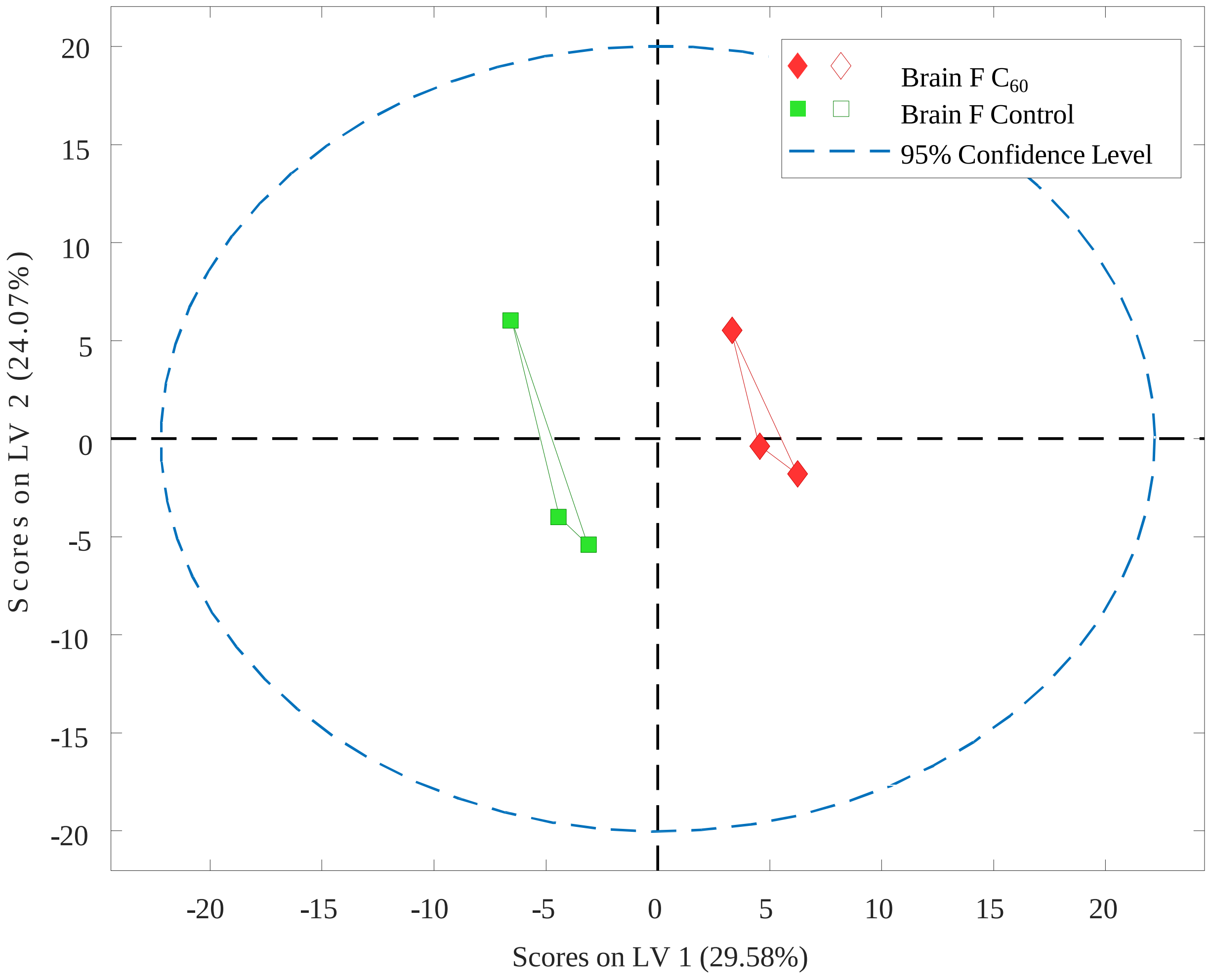

B]

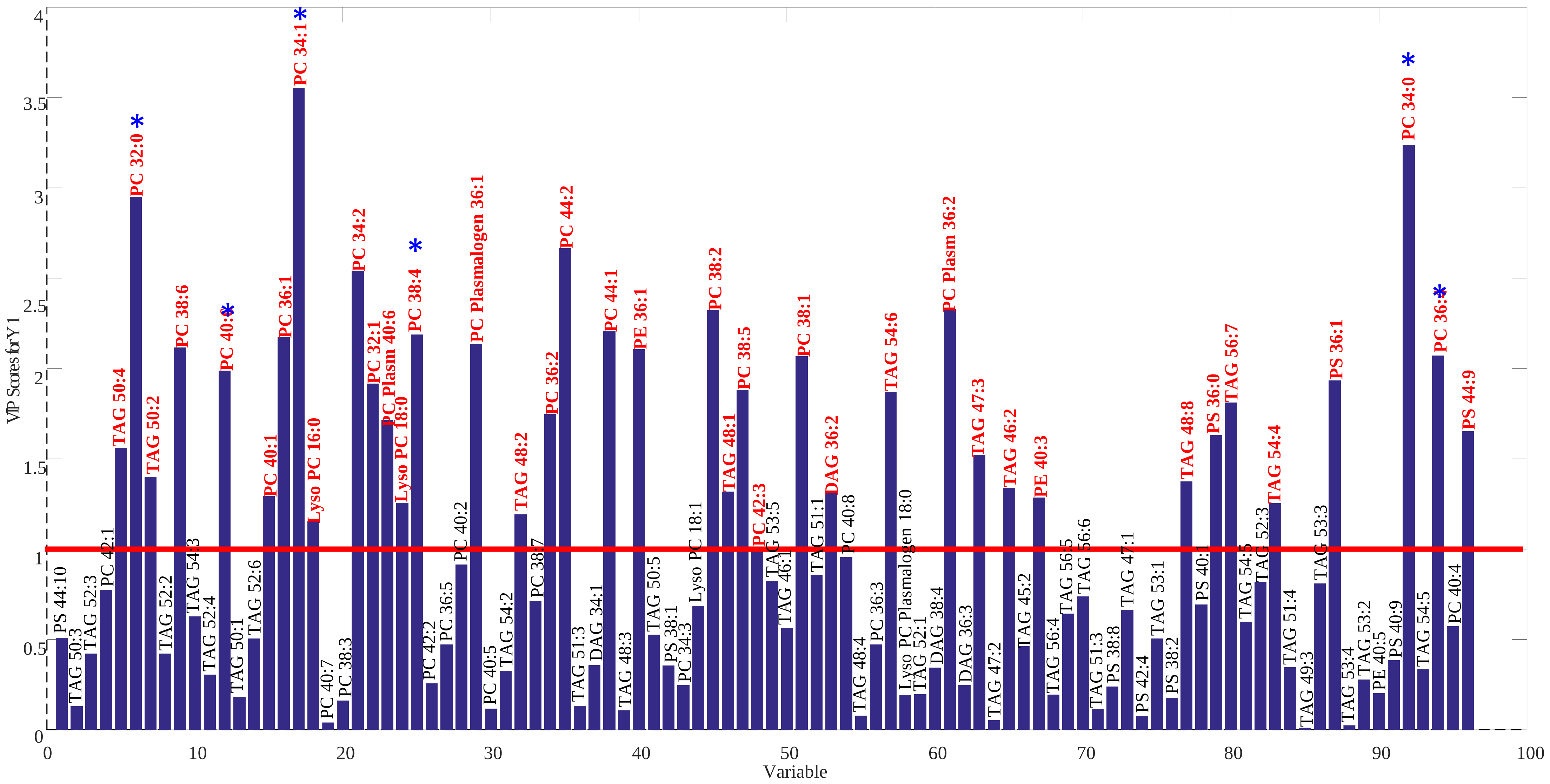

Table S1. Potential biomarkers for lipid disruption in distinct tissues of zebrafish exposed to carbon-based nanoparticles. Lipid species in stressed samples showing significant down- or up- regulation respect to controls were determined by PLS-DA analyses (VIP > 1) and further two-sample Student's *t*-Test (*P* < 0.05). (See Section 2.5. in the manuscript).

Lipid specie	Lipid classification	Fold-change[a]	*p*-values[b]	Up(+)/down(-) regulation
**BRAIN FEMALE**				
**C_{60}-treatment**				
PC 32:0	Glycerophospholipid	1.27	0.00433	+
PC 34:0	Glycerophospholipid	1.24	0.04483	+
PC 34:1	Glycerophospholipid	1.24	0.00070	+
PC 36:4	Glycerophospholipid	2.52	0.03353	+
PC 38:4	Glycerophospholipid	2.18	0.04557	+
PC 40:6	Glycerophospholipid	1.21	0.02364	+
**SWCNT-treatment**				
PC 34:1	Glycerophospholipid	1.23	0.02501	+
**Short MWCNT-treatment**				
PC 32:0	Glycerophospholipid	1.24	0.02088	+
PC 34:0	Glycerophospholipid	1.20	0.01347	+
PC 34:1	Glycerophospholipid	1.26	0.00054	+
Plasmalogen PC 36:1	Glycerophospholipid	1.21	0.03517	+
PS 44:9	Glycerophospholipid	1.67	0.04574	-
TAG 52:3	Glycerolipid	1.90	0.04651	+
TAG 52:4	Glycerolipid	1.67	0.03786	+
TAG 54:3	Glycerolipid	1.38	0.04367	+
**Long MWCNT-treatment**				
PC 34:1	Glycerophospholipid	1.13	0.02182	+
Plasmalogen PC 36:1	Glycerophospholipid	1.28	0.00584	+
TAG 54:2	Glycerolipid	1.50	0.02358	-
TAG 54:3	Glycerolipid	1.59	0.04319	-
**BRAIN MALE**				
**C_{60}-treatment**				
PC 32:0	Glycerophospholipid	1.65	0.03755	-
PC 32:1	Glycerophospholipid	1.27	0.04428	-
PC 34:0	Glycerophospholipid	1.41	0.00615	-
PC 34:1	Glycerophospholipid	1.36	0.01910	-
PC 36:1	Glycerophospholipid	1.42	0.00498	-
PC 40:1	Glycerophospholipid	1.58	0.02230	-
PC 40:6	Glycerophospholipid	1.41	0.02480	-
PE 36:1	Glycerophospholipid	1.23	0.01975	-
TAG 52:3	Glycerolipid	4.92	0.00453	-
**SWCNT-treatment**				
PC 32:0	Glycerophospholipid	1.07	0.04442	+
PC 36:2	Glycerophospholipid	1.60	0.00027	+
PC 38:2	Glycerophospholipid	1.40	0.01532	+
PC 38:6	Glycerophospholipid	1.28	0.02924	+
PC 42:1	Glycerophospholipid	2.06	0.02671	+
Plasmalogen PC 36:1	Glycerophospholipid	1.49	0.00824	+
PE 36:1	Glycerophospholipid	1.22	0.00659	+
TAG 48:2	Glycerolipid	1.34	0.03339	+
TAG 50:1	Glycerolipid	1.20	0.00340	+
TAG 52:3	Glycerolipid	1.73	0.03941	+
TAG 54:3	Glycerolipid	2.05	0.01670	+

**Short MWCNT-treatment**				
PC 36:1	Glycerophospholipid	3.32	0.00028	-
PC 36:4	Glycerophospholipid	9.28	0.04385	-
PC 38:2	Glycerophospholipid	4.61	0.00746	-
PC 38:6	Glycerophospholipid	5.40	0.00251	-
PC 42:2	Glycerophospholipid	4.40	0.00042	-
Plasmalogen PC 36:1	Glycerophospholipid	3.08	0.00260	-
Plasmalogen PC 36:2	Glycerophospholipid	2.76	0.00565	-
PE 36:1	Glycerophospholipid	5.43	0.00917	-
PE 40:5	Glycerophospholipid	10.77	0.00033	-
TAG 48:2	Glycerolipid	2.97	0.02315	-
TAG 51:2	Glycerolipid	1.30	0.00391	-
TAG 56:7	Glycerolipid	1.94	0.00459	-
**Long MWCNT-treatment**				
PC 34:0	Glycerophospholipid	1.19	0.00390	+
PC 34:1	Glycerophospholipid	1.25	0.01552	+
PC 36:1	Glycerophospholipid	1.20	0.04961	+
PC 38:2	Glycerophospholipid	1.5	0.02783	+
PC 38:4	Glycerophospholipid	1.82	0.04112	+
PC 40:1	Glycerophospholipid	2.27	0.04875	+
PC 40:6	Glycerophospholipid	1.16	0.01137	+
PC 42:1	Glycerophospholipid	2.17	0.00094	+
PC 42:2	Glycerophospholipid	1.39	0.49028	+
PC 44:2	Glycerophospholipid	3.37	0.00698	+
Plasmalogen PC 36:1	Glycerophospholipid	1.56	0.00654	+
PS 40:9	Glycerophospholipid	2.42	0.04645	+
PS 44:9	Glycerophospholipid	1.68	0.02099	+
TAG 48:2	Glycerolipid	1.43	0.00403	+
TAG 50:1	Glycerolipid	1.19	0.02154	+
TAG 51:1	Glycerolipid	1.32	0.03486	+
TAG 52:4	Glycerolipid	2.21	0.00919	+
TAG 54:2	Glycerolipid	1.37	0.03118	+
TAG 54:5	Glycerolipid	3.09	0.01760	+
**GONAD FEMALE**				
**C_{60}-treatment**				
PC 38:4	Glycerophospholipid	1.32	0.00941	+
PC 40:4	Glycerophospholipid	1.67	0.01783	+
PC 40:5	Glycerophospholipid	1.61	0.01354	+
Plasmalogen PC 36:1	Glycerophospholipid	1.28	0.04628	+
Plasmalogen PC 36:2	Glycerophospholipid	1.05	0.00628	-
Plasmalogen PC 40:6	Glycerophospholipid	1.28	0.01150	+
PE 36:1	Glycerophospholipid	1.14	0.01093	+
TAG 46:1	Glycerolipid	1.41	0.04471	+
TAG 48:1	Glycerolipid	1.71	0.04178	+
TAG 52:2	Glycerolipid	1.22	0.04604	-
TAG 54:4	Glycerolipid	1.34	0.01140	-
TAG 54:5	Glycerolipid	1.60	0.01531	-
TAG 54:6	Glycerolipid	1.15	0.02472	-
TAG 56:6	Glycerolipid	1.38	0.02793	-
**SWCNT-treatment**				
PC 32:0	Glycerophospholipid	1.65	0.01017	-
PC 34:3	Glycerophospholipid	4.15	0.02982	-
PC 36:5	Glycerophospholipid	5.10	0.01138	-
PC 38:3	Glycerophospholipid	2.51	0.00188	-
PC 38:4	Glycerophospholipid	1.71	0.00623	-
PC 38:7	Glycerophospholipid	5.25	0.01556	-
PC 40:5	Glycerophospholipid	1.82	0.00681	-
PC 40:6	Glycerophospholipid	1.91	0.00395	-
PC 40:7	Glycerophospholipid	2.00	0.01548	-
PC 40:8	Glycerophospholipid	4.18	0.02038	-

Plasmalogen PC 36:1	Glycerophospholipid	1.82	0.00513	-
Plasmalogen PC 36:2	Glycerophospholipid	2.33	0.00619	-
Plasmalogen PC 40:6	Glycerophospholipid	1.76	0.00524	-
PE 36:1	Glycerophospholipid	1.81	0.00852	-
PS 36:0	Glycerophospholipid	1.99	0.02070	-
PS 38:8	Glycerophospholipid	1.73	0.00401	-
TAG 48:1	Glycerolipid	1.85	0.02626	-
TAG 48:2	Glycerolipid	4.08	0.00712	-
TAG 50:2	Glycerolipid	3.91	0.00813	-
TAG 50:4	Glycerolipid	6.22	0.00036	-
TAG 50:5	Glycerolipid	4.27	0.01845	-
TAG 51:1	Glycerolipid	2.64	0.00747	-
TAG 52:6	Glycerolipid	9.47	0.00125	-
TAG 53:4	Glycerolipid	10.27	0.03470	-
TAG 54:3	Glycerolipid	7.24	0.00139	-
TAG 54:5	Glycerolipid	4.85	0.00121	-
**Short MWCNT-treatment**				
PC 38:4	Glycerophospholipid	1.35	0.01163	+
PC 38:5	Glycerophospholipid	1.20	0.01885	+
PC 38:7	Glycerophospholipid	1.72	0.03684	+
PC 40:4	Glycerophospholipid	1.97	0.02234	+
PC 40:5	Glycerophospholipid	1.5	0.02276	+
Plasmalogen PC 36:1	Glycerophospholipid	1.34	0.02652	+
PE 36:1	Glycerophospholipid	1.44	0.03396	+
PS 40:9	Glycerophospholipid	1.22	0.03952	+
TAG 48:1	Glycerolipid	1.62	0.00595	+
TAG 48:2	Glycerolipid	1.58	0.01842	+
TAG 50:5	Glycerolipid	1.63	0.00283	+
TAG 52:3	Glycerolipid	1.16	0.01998	+
TAG 54:2	Glycerolipid	1.20	0.04159	-
**Long MWCNT-treatment**				
PC 38:7	Glycerophospholipid	1.57	0.01038	-
PC 34:0	Glycerophospholipid	1.17	0.03019	-
PC 34:3	Glycerophospholipid	1.38	0.01775	-
PC 38:3	Glycerophospholipid	1.32	0.01569	-
PC 40:8	Glycerophospholipid	1.35	0.02246	-
Plasmalogen PC 36:2	Glycerophospholipid	1.25	0.03679	-
PE 40:3	Glycerophospholipid	1.25	0.01265	-
PS 44:9	Glycerophospholipid	1.47	0.03745	-
PS 44:10	Glycerophospholipid	1.53	0.04856	-
TAG 46:1	Glycerolipid	1.66	0.02385	+
TAG 48:1	Glycerolipid	2.19	0.01589	+
TAG 48:2	Glycerolipid	1.71	0.01393	+
TAG 48:8	Glycerolipid	1.86	0.03248	-
TAG 52:2	Glycerolipid	1.30	0.03318	-
TAG 53:2	Glycerolipid	1.38	0.03547	-
TAG 53:3	Glycerolipid	2.08	0.01287	-
TAG 54:3	Glycerolipid	1.50	0.01735	-
TAG 54:4	Glycerolipid	1.67	0.01082	-
TAG 54:5	Glycerolipid	1.60	0.01728	-
TAG 54:6	Glycerolipid	1.43	0.03137	-
TAG 56:5	Glycerolipid	2.20	0.00394	-
TAG 56:7	Glycerolipid	1.60	0.03255	-
**GONAD MALE**				
**C_{60}-treatment**				
PC 34:3	Glycerophospholipid	1.38	0.01775	-
PS 44:9	Glycerophospholipid	1.47	0.03745	-
PS 44:10	Glycerophospholipid	1.53	0.04856	-
TAG 48:1	Glycerolipid	2.19	0.01588	+
TAG 48:2	Glycerolipid	1.71	0.01392	+
TAG 46:1	Glycerolipid	1.66	0.02385	+

TAG 52:2	Glycerolipid	1.30	0.03318	-
TAG 53:2	Glycerolipid	1.38	0.03547	-
TAG 53:3	Glycerolipid	2.08	0.01287	-
TAG 54:3	Glycerolipid	1.50	0.01735	-
TAG 54:4	Glycerolipid	1.67	0.01082	-
TAG 54:5	Glycerolipid	1.60	0.01725	-
TAG 54:6	Glycerolipid	1.43	0.03137	+
TAG 56:5	Glycerolipid	2.19	0.00394	-
**SWCNT-treatment**				
PC 34:3	Glycerophospholipid	1.38	0.01775	-
PC 38:7	Glycerophospholipid	1.57	0.01038	-
PS 44:9	Glycerophospholipid	1.47	0.03745	-
TAG 46:1	Glycerolipid	1.66	0.02385	+
TAG 48:1	Glycerolipid	2.19	0.01589	+
TAG 48:2	Glycerolipid	1.71	0.01393	+
TAG 48:8	Glycerolipid	1.86	0.03248	-
TAG 52:2	Glycerolipid	1.30	0.03318	-
TAG 54:3	Glycerolipid	1.50	0.01736	-
TAG 54:4	Glycerolipid	1.67	0.01082	-
TAG 54:5	Glycerolipid	2.24	0.00261	-
TAG 54:6	Glycerolipid	1.44	0.03137	-
TAG 56:5	Glycerolipid	2.20	0.00393	-
TAG 56:7	Glycerolipid	1.60	0.03255	-
**Short MWCNT-treatment**				
PC 34:1	Glycerophospholipid	7.67	0.00705	-
PC 34:2	Glycerophospholipid	1.79	0.02747	-
PC 36:4	Glycerophospholipid	2.89	0.01655	-
PC 38:5	Glycerophospholipid	3.48	0.01722	-
PC 38:6	Glycerophospholipid	7.24	0.00164	-
TAG 45:2	Glycerolipid	2.97	0.02125	-
TAG 46:1	Glycerolipid	1.45	0.01502	-
TAG 47:2	Glycerolipid	1.60	0.00619	-
TAG 47:3	Glycerolipid	2.89	0.01534	-
TAG 48:1	Glycerolipid	1.43	0.02177	-
TAG 51:3	Glycerolipid	1.12	0.04120	-
TAG 52:3	Glycerolipid	1.45	0.04158	-
TAG 54:5	Glycerolipid	1.49	0.04263	-
TAG 56:6	Glycerolipid	2.63	0.00001	-
TAG 56:7	Glycerolipid	3.15	0.03554	-
**Long MWCNT-treatment**				
PC 32:0	Glycerophospholipid	1.89	0.01584	+
PC 34:1	Glycerophospholipid	1.75	0.02775	+
PC 34:2	Glycerophospholipid	2.02	0.01267	+
PC 36:2	Glycerophospholipid	3.87	0.02230	+
PC 36:3	Glycerophospholipid	3.08	0.00047	+
PC 36:5	Glycerophospholipid	3.50	0.02086	+
PC 38:4	Glycerophospholipid	3.64	0.00095	+
PC 38:5	Glycerophospholipid	1.95	0.00705	+
PC 38:6	Glycerophospholipid	1.55	0.02194	+
PC 40:6	Glycerophospholipid	2.91	0.00048	+
Plasmalogen PC 40:6	Glycerophospholipid	2.73	0.00059	+
PS 44:9	Glycerophospholipid	1.25	0.00657	+
TAG 48:8	Glycerolipid	1.66	0.04382	-
TAG 52:3	Glycerolipid	1.20	0.01093	+
TAG 54:3	Glycerolipid	1.38	0.00789	+
TAG 54:5	Glycerolipid	1.36	0.01044	+
TAG 54:6	Glycerolipid	1.34	0.04136	+
TAG 56:7	Glycerolipid	1.20	0.03363	-
**GASTRO-INTESTINAL TRACT FEMALE**				
**C_{60}-treatment**				

32:0	Glycerophospholipid	1.59	0.01646	+
PC 34:1	Glycerophospholipid	1.49	0.01489	+
PC 36:2	Glycerophospholipid	3.04	0.00327	+
PC 38:5	Glycerophospholipid	3.33	0.01694	+
Plasmalogen PC 36:1	Glycerophospholipid	2.05	0.01539	+
Plasmalogen PC 36:2	Glycerophospholipid	1.74	0.04366	+
PS 36:0	Glycerophospholipid	2.69	0.01864	+
PS 36:1	Glycerophospholipid	5.50	0.01236	+
PS 38:1	Glycerophospholipid	3.32	0.00129	+
PS 40:1	Glycerophospholipid	3.48	0.00475	-
PS 40:9	Glycerophospholipid	2.39	0.01905	+
PS 42:4	Glycerophospholipid	2.97	0.02799	+
PS 44:10	Glycerophospholipid	2.20	0.02911	+
TAG 54:2	Glycerolipid	2.38	0.00423	+
DAG 34:1	Glycerolipid	1.78	0.02749	-
TAG 53:5	Glycerolipid	2.80	0.00608	+
TAG 46:1	Glycerolipid	1.90	0.03871	+
TAG 47:1	Glycerolipid	2.91	0.03802	+
TAG 49:3	Glycerolipid	3.67	0.00569	+
TAG 50:3	Glycerolipid	3.96	0.01189	+
TAG 50:5	Glycerolipid	3.16	0.00987	+
TAG 51:2	Glycerolipid	2.34	0.04958	+
TAG 51:3	Glycerolipid	3.65	0.07862	+
TAG 51:4	Glycerolipid	5.02	0.01363	+
TAG 52:2	Glycerolipid	1.85	0.03951	+
TAG 52:3	Glycerolipid	12.98	0.01306	+
TAG 52:4	Glycerolipid	2.28	0.01526	+
TAG 52:6	Glycerolipid	6.71	0.03478	+
TAG 53:3	Glycerolipid	2.19	0.03406	+
TAG 53:4	Glycerolipid	4.67	0.01247	+
TAG 54:4	Glycerolipid	2.36	0.00968	+
TAG 54:5	Glycerolipid	2.05	0.03297	-
TAG 54:6	Glycerolipid	1.91	0.02795	+
TAG 56:5	Glycerolipid	6.16	0.00343	+
TAG 56:6	Glycerolipid	3.71	0.01635	+
**SWCNT-treatment**				
PC 34:1	Glycerophospholipid	1.71	0.02575	+
PC 36:1	Glycerophospholipid	1.46	0.39146	+
PC 40:4	Glycerophospholipid	3.53	0.00142	+
PC 40:5	Glycerophospholipid	4.25	0.01664	+
Plasmalogen PC 36:1	Glycerophospholipid	1.78	0.01726	+
Plasmalogen PC 36:2	Glycerophospholipid	1.97	0.01636	+
PS 36:1	Glycerophospholipid	8.62	0.02691	+
PS 38:1	Glycerophospholipid	2.14	0.00478	+
PS 38:8	Glycerophospholipid	3.49	0.06795	-
TAG 52:3	Glycerolipid	10.24	0.00050	+
TAG 52:4	Glycerolipid	1.42	0.00948	+
TAG 54:3	Glycerolipid	3.01	0.00417	+
TAG 54:4	Glycerolipid	1.60	0.01738	+
TAG 54:5	Glycerolipid	2.40	0.01902	-
TAG 54:6	Glycerolipid	1.36	0.00410	+
TAG 56:5	Glycerolipid	4.74	0.00167	+
TAG 56:6	Glycerolipid	3.88	0.00384	+
TAG 56:7	Glycerolipid	3.26	0.00811	+
DAG 34:1	Glycerolipid	1.45	0.03137	-
**Short MWCNT-treatment**				
PC 34:1	Glycerophospholipid	1.96	0.03000	-
PC 36:1	Glycerophospholipid	2.81	0.01082	-
PC 40:4	Glycerophospholipid	1.43	0.00448	-
Plasmalogen PC 36:1	Glycerophospholipid	1.65	0.00316	-
Plasmalogen PC 36:2	Glycerophospholipid	2.52	0.02248	-
PS 36:1	Glycerophospholipid	2.48	0.02339	+
PS 38:8	Glycerophospholipid	5.45	0.04351	-

PS 40:1	Glycerophospholipid	9.20	0.04185	-
TAG 50:3	Glycerolipid	1.39	0.03074	-
TAG 52:3	Glycerolipid	4.15	0.02796	+
TAG 52:4	Glycerolipid	1.76	0.00970	-
TAG 54:5	Glycerolipid	2.07	0.02286	-
DAG 34:1	Glycerolipid	1.81	0.01293	-
TAG 54:6	Glycerolipid	1.47	0.01632	-
TAG 56:5	Glycerolipid	2.42	0.02334	+
TAG 56:6	Glycerolipid	2.54	0.01408	+
TAG 56:7	Glycerolipid	2.06	0.01472	+
**Long MWCNT-treatment**				
PC 32:0	Glycerophospholipid	1.36	0.04101	-
PC 40:4	Glycerophospholipid	2.79	0.01009	-
Plasmalogen PC 36:2	Glycerophospholipid	2.38	0.01989	-
PS 38:1	Glycerophospholipid	3.50	0.00037	+
PS 38:8	Glycerophospholipid	5.67	0.04532	-
PS 40:9	Glycerophospholipid	2.00	0.02944	+
PS 44:9	Glycerophospholipid	2.32	0.03354	+
PS 44:10	Glycerophospholipid	2.13	0.01615	+
TAG 46:1	Glycerolipid	1.99	0.02461	+
TAG 47:1	Glycerolipid	3.34	0.03561	+
TAG 48:2	Glycerolipid	1.54	0.03932	+
TAG 49:3	Glycerolipid	3.02	0.00902	+
TAG 50:1	Glycerolipid	1.95	0.00810	+
TAG 50:2	Glycerolipid	1.83	0.03918	+
TAG 50:3	Glycerolipid	4.89	0.04333	+
TAG 50:4	Glycerolipid	2.91	0.00496	+
TAG 51:2	Glycerolipid	2.39	0.00818	+
TAG 52:3	Glycerolipid	10.73	0.00073	+
TAG 52:4	Glycerolipid	2.24	0.00008	+
TAG 52:6	Glycerolipid	3.13	0.02218	+
TAG 53:3	Glycerolipid	2.11	0.00316	+
TAG 53:4	Glycerolipid	4.30	0.00081	+
TAG 54:3	Glycerolipid	4.44	0.01160	+
TAG 54:4	Glycerolipid	2.26	0.00465	+
TAG 54:5	Glycerolipid	2.03	0.00199	-
TAG 54:6	Glycerolipid	1.70	0.00022	+
TAG 56:5	Glycerolipid	5.52	0.00061	+
TAG 56:6	Glycerolipid	2.81	0.00618	+
TAG 56:7	Glycerolipid	2.76	0.00657	+
**GASTRO-INTESTINAL TRACT MALE**				
**C_{60}-treatment**				
PC 36:4	Glycerophospholipid	1.95	0.01961	-
PC 38:4	Glycerophospholipid	1.66	0.02040	-
PC 38:5	Glycerophospholipid	1.80	0.02324	-
PC 38:6	Glycerophospholipid	1.80	0.02874	-
PC 40:6	Glycerophospholipid	1.81	0.02861	-
PC 40:7	Glycerophospholipid	1.96	0.00392	-
Lyso PC 16:0	Glycerophospholipid	7.75	0.00313	-
Lyso PC 18:0	Glycerophospholipid	4.87	0.03126	-
Lyso PC 18:1	Glycerophospholipid	9.49	0.00317	-
Plasmalogen PC 40:6	Glycerophospholipid	1.59	0.01805	-
Lyso Plasmalogen PC	Glycerophospholipid	3.85	0.03956	-
18:0	Glycerophospholipid	1.67	0.01297	-
PS 36:1	Glycerophospholipid	1.19	0.03138	-
PS 38:1	Glycerophospholipid	2.78	0.03726	+
PS 38:8	Glycerophospholipid	1.32	0.02609	-
TAG 48:2	Glycerolipid	1.24	0.03685	-
TAG 50:4	Glycerolipid	1.22	0.03497	-
TAG 53:3	Glycerolipid	1.23	0.02224	+
TAG 54:2	Glycerolipid	1.26	0.01678	-

SWCNT-treatment

PC 34:1	Glycerophospholipid	2.31	0.00227	-
PC 34:2	Glycerophospholipid	3.36	0.03373	-
PC 36:1	Glycerophospholipid	1.91	0.03183	-
PC 36:3	Glycerophospholipid	2.33	0.00074	-
PC 36:4	Glycerophospholipid	6.61	0.00505	-
PC 36:5	Glycerophospholipid	12.06	0.04318	-
PC 38:4	Glycerophospholipid	6.10	0.00786	-
PC 38:5	Glycerophospholipid	6.24	0.00820	-
PC 38:6	Glycerophospholipid	5.66	0.01963	-
PC 40:6	Glycerophospholipid	6.49	0.00893	-
PC 40:7	Glycerophospholipid	3.78	0.00209	-
Lyso PC 16:0	Glycerophospholipid	4.96	0.01989	-
Lyso PC 18:0	Glycerophospholipid	4.67	0.01783	-
Lyso PC 18:1	Glycerophospholipid	2.20	0.02408	-
Plasmalogen PC 40:6	Glycerophospholipid	3.71	0.00248	-
Lyso PlasmalogenPC 18:0	Glycerophospholipid	2.84	0.03156	-
PE 40:3	Glycerophospholipid	2.91	0.00497	-
PE 40:5	Glycerophospholipid	10.03	0.00210	-
PS 36:1	Glycerophospholipid	14.40	0.00854	-
PS 38:1	Glycerophospholipid	1.19	0.04901	-
PS 44:9	Glycerophospholipid	1.52	0.00047	+
PS 44:10	Glycerophospholipid	1.29	0.03589	+
TAG 48:1	Glycerophospholipid	1.34	0.00457	-
TAG 48:2	Glycerolipid	1.48	0.04321	-
TAG 50:3	Glycerolipid	1.21	0.02779	-
TAG 54:2	Glycerolipid	1.44	0.01904	+
TAG 54:3	Glycerolipid	1.54	0.00677	+
TAG 54:4	Glycerolipid	1.22	0.04494	+
TAG 54:5	Glycerolipid	1.06	0.01246	+
TAG 54:6	Glycerolipid	1.09	0.00416	-
TAG 56:5	Glycerolipid	1.65	0.02853	+
TAG 56:6	Glycerolipid	1.60	0.04519	+

Short MWCNT-treatment

PC 34:1	Glycerophospholipid	27.47	0.00229	-
PC 34:2	Glycerophospholipid	4.43	0.03245	-
PC 36:1	Glycerophospholipid	3.01	0.00792	-
PC 36:3	Glycerophospholipid	3.84	0.00369	-
PC 36:4	Glycerophospholipid	8.10	0.00451	-
PC 38:4	Glycerophospholipid	13.32	0.01111	-
PC 38:5	Glycerophospholipid	7.02	0.01184	-
PC 38:6	Glycerophospholipid	11.44	0.01856	-
PC 40:6	Glycerophospholipid	6.46	0.00925	-
PC 40:7	Glycerophospholipid	3.77	0.00215	-
Lyso PC 16:0	Glycerophospholipid	4.88	0.02344	-
Lyso PC 18:0	Glycerophospholipid	4.60	0.01923	-
Lyso PC 18:1	Glycerophospholipid	1.97	0.03230	-
Plasmalogen PC 40:6	Glycerophospholipid	7.52	0.01188	-
Lyso PlasmalogenPC 18:0	Glycerophospholipid	2.59	0.03737	-
PE 40:3	Glycerophospholipid	3.67	0.00305	-
PE 40:5	Glycerophospholipid	2.96	0.03828	-
PS 36:1	Glycerophospholipid	3.39	0.00534	-
PS 38:1	Glycerophospholipid	1.98	0.00244	-
PS 40:9	Glycerophospholipid	14.79	0.01355	-
PS 44:9	Glycerophospholipid	6.81	0.02182	-
PS 44:10	Glycerophospholipid	4.34	0.00105	-
TAG 47:3	Glycerolipid	1.32	0.01048	-
TAG 48:1	Glycerolipid	2.46	0.00168	-
TAG 48:2	Glycerolipid	5.68	0.00313	-
TAG 50:3	Glycerolipid	6.21	0.00050	-
TAG 50:4	Glycerolipid	5.92	0.00426	-
TAG 51:2	Glycerolipid	3.96	0.00075	-
TAG 52:2	Glycerolipid	3.09	0.01463	-
TAG 52:6	Glycerolipid	9.32	0.00982	-

TAG 53:1	Glycerolipid	2.94	0.04794	-
TAG 53:2	Glycerolipid	3.30	0.01236	-
TAG 53:5	Glycerolipid	3.07	0.00719	-
TAG 54:2	Glycerolipid	2.60	0.00590	-
TAG 54:3	Glycerolipid	3.76	0.00738	-
TAG 54:4	Glycerolipid	4.22	0.00156	-
TAG 54:6	Glycerolipid	4.00	0.00026	-
TAG 56:5	Glycerolipid	6.36	0.01149	-
TAG 56:6	Glycerolipid	2.44	0.02166	-
TAG 56:7	Glycerolipid	3.80	0.00316	-
**Long MWCNT-treatment**				
PC 36:3	Glycerophospholipid	1.36	0.00763	-
PC 38:5	Glycerophospholipid	1.50	0.01597	-
Lyso PC 16:0	Glycerophospholipid	1.91	0.00313	+
Lyso PC 18:0	Glycerophospholipid	3.90	0.00550	-
Lyso PC 18:1	Glycerophospholipid	3.80	0.00700	-
Plasmalogen PC 36:1	Glycerophospholipid	3.81	0.00366	+
Plasmalogen PC 36:2	Glycerophospholipid	2.88	0.00017	+
PS 36:1	Glycerophospholipid	1.25	0.04929	-
PS 38:1	Glycerophospholipid	1.24	0.02154	+
PS 38:2	Glycerophospholipid	3.68	0.00346	+
PS 42:4	Glycerophospholipid	2.12	0.00304	+
PS 44:9	Glycerophospholipid	2.26	0.00078	+
PS 44:10	Glycerophospholipid	3.54	0.00574	-
TAG 46:1	Glycerolipid	2.02	0.00927	+
TAG 46:2	Glycerolipid	4.01	0.00226	+
TAG 47:1	Glycerolipid	3.05	0.03412	+
TAG 47:2	Glycerolipid	3.14	0.00454	+
TAG 48:4	Glycerolipid	3.68	0.00601	+
TAG 48:8	Glycerolipid	1.95	0.02620	+
TAG 50:1	Glycerolipid	1.53	0.01396	+
TAG 50:2	Glycerolipid	1.69	0.00846	+
TAG 50:3	Glycerolipid	1.27	0.00192	+
TAG 50:4	Glycerolipid	1.21	0.03187	+
TAG 50:5	Glycerolipid	1.65	0.00774	+
TAG 51:1	Glycerolipid	1.40	0.03999	+
TAG 51:2	Glycerolipid	1.39	0.02665	+
TAG 52:2	Glycerolipid	2.09	0.00846	+
TAG 52:3	Glycerolipid	2.18	0.04763	+
TAG 52:4	Glycerolipid	1.56	0.00083	+
TAG 52:6	Glycerolipid	1.82	0.02020	+
TAG 53:1	Glycerolipid	2.04	0.04333	+
TAG 53:2	Glycerolipid	2.10	0.01378	+
TAG 53:3	Glycerolipid	1.44	0.02969	+
TAG 53:4	Glycerolipid	2.01	0.04109	+
TAG 53:5	Glycerolipid	1.31	0.03052	+
TAG 54:2	Glycerolipid	2.35	0.01541	+
TAG 54:3	Glycerolipid	2.43	0.01820	+
TAG 54:4	Glycerolipid	1.84	0.00142	+
TAG 54:5	Glycerolipid	1.56	0.00559	+
TAG 54:6	Glycerolipid	1.43	0.00271	+
TAG 56:5	Glycerolipid	1.97	0.00279	+
TAG 56:6	Glycerolipid	2.16	0.00177	+
TAG 56:7	Glycerolipid	1.66	0.00239	+
DAG 34:1	Glycerolipid	3.71	0.00020	+
DAG 36:2	Glycerolipid	3.87	0.00292	+
DAG 36:3	Glycerolipid	2.90	0.02318	+

[a]Fold change- mean of stressed-samples/mean of control samples.
[b]*p*-value- Significance levels for the two-sample Student's *t*-Test were computed for assessing the significance between stressed and control samples.

Table S2. Elemental composition of glycerophospholipid and glycerolipid species found in brain, gonad and gastro-intestinal tract tissues of zebrafish, calculated by mass accuracy within error of 10 ppm, with atom constraints and with $-0.5 \leq DBE \leq 15.0$. DBE: double-bond equivalent. Elemental composition of PC, Plasmalogen PC, Lyso PC, Lyso Plasmalogen PC and PE refer to the $[M+H]^{+}$ ions whereas TAG, DAG and PS refer to ammonium adducts $[M + NH_4]^{+}$. Lipid species were detected under ESI (+) using an UHPLC system coupled to a TOF analyzer with an Acquity UPLC BEH C_8 column (1.7 µm particle size, 100 mm x 2.1 mm).

GLYCEROPHOSPHOLIPIDS						
**Lipid subclass**	**Lipid specie**	**Measured mass (Da)**	**Elemental composition**	**Calculated mass (Da)**	**Error (ppm)**	**DBE**
**PC**						
	32:0	734.5750	$C_{40}H_{81}NO_8P$	734.5694	7.6	1.5
	32:1	732.5600	$C_{40}H_{79}NO_8P$	732.5538	8.5	2.5
	34:0	762.6013	$C_{42}H_{85}NO_8P$	762.6007	0.8	1.5
	34:1	760.5900	$C_{42}H_{83}NO_8P$	760.5851	6.4	2.5
	34:2	758.5760	$C_{42}H_{81}NO_8P$	758.5694	8.7	3.5
	34:3	756.5613	$C_{42}H_{79}NO_8P$	756.5538	9.9	4.5
	36:1	788.6234	$C_{44}H_{87}NO_8P$	788.6164	8.9	2.5
	36:2	786.6085	$C_{44}H_{85}NO_8P$	786.6007	9.9	3.5
	36:3	784.5890	$C_{44}H_{83}NO_8P$	784.5851	5.0	4.5
	36:4	782.5760	$C_{44}H_{81}NO_8P$	782.5694	8.4	5.5
	36:5	780.5600	$C_{44}H_{79}NO_8P$	780.5538	7.9	6.5
	38:1	816.6543	$C_{46}H_{91}NO_8P$	816.6478	8.0	2.5
	38:2	814.6400	$C_{46}H_{89}NO_8P$	814.6320	9.8	3.5
	38:3	812.6200	$C_{46}H_{87}NO_8P$	812.6164	4.4	4.5
	38:4	810.6057	$C_{46}H_{85}NO_8P$	810.6007	6.2	5.5
	38:5	808.5897	$C_{46}H_{83}NO_8P$	808.5851	5.7	6.5
	38:6	806.5620	$C_{46}H_{81}NO_8P$	806.5694	-9.2	7.5
	38:7	804.5600	$C_{46}H_{79}NO_8P$	804.5537	7.8	8.5
	40:1	844.6864	$C_{48}H_{95}NO_8P$	844.6790	8.8	1.5
	40:2	842.6706	$C_{48}H_{93}NO_8P$	842.6633	8.7	3.5
	40:4	838.6339	$C_{48}H_{89}NO_8P$	838.6320	2.3	5.5
	40:5	836.6189	$C_{48}H_{87}NO_8P$	836.6164	3.0	6.5
	40:6	834.6052	$C_{48}H_{85}NO_8P$	834.6007	5.4	7.5
	40:7	832.5900	$C_{48}H_{83}NO_8P$	832.5850	6.0	8.5
	40:8	830.5733	$C_{48}H_{81}NO_8P$	830.5694	4.7	9.5
	42:1	872.7190	$C_{50}H_{99}NO_8P$	872.7102	10.0	2.5
	42:2	870.7000	$C_{50}H_{97}NO_8P$	870.6946	6.2	3.5
	42:3	868.6840	$C_{50}H_{95}NO_8P$	868.6790	5.8	4.5
	44:1	900.7500	$C_{52}H_{103}NO_8P$	900.7415	9.4	1.5
	44:2	898.7349	$C_{52}H_{101}NO_8P$	898.7259	10.0	2.5
**Plasmalogen PC**						
	36:1	774.6292	$C_{44}H_{89}NO_7P$	774.6371	-10.0	1.5
	36:2	772.6143	$C_{44}H_{87}NO_7P$	772.6215	-9.4	2.5
	40:6	820.6140	$C_{48}H_{87}NO_7P$	820.6215	-9.1	6.5
**Lyso PC**						
	16:0	496.3390	$C_{24}H_{51}NO_7P$	496.3398	1.6	0.5
	18:0	524.3671	$C_{26}H_{55}NO_7P$	524.3711	7.6	0.5
	18:1	522.3540	$C_{26}H_{53}NO_7P$	522.3554	2.7	1.5

**Lyso plasmalogen PC**					
18:0	510.3877	$C_{26}H_{57}NO_6P$	510.3918	-8.0	-0.5
**PE**					
36:1	746.5760	$C_{41}H_{81}NO_8P$	746.5694	8.8	2.5
40:3	796.5916	$C_{45}H_{83}NO_8P$	796.5851	8.2	5.5
40:5	792.5607	$C_{45}H_{79}NO_8P$	792.5538	8.7	7.5
**PS**					
36:0	809.5970	$C_{42}H_{86}N_2O_{10}P$	809.6015	5.5	1.5
36:1	807.5830	$C_{42}H_{84}N_2O_{10}P$	807.5858	3.5	2.5
38:1	835.7672	$C_{44}H_{88}N_2O_{10}P$	835.6172	5.4	2.5
38:2	833.6100	$C_{44}H_{86}N_2O_{10}P$	833.6015	10.0	3.5
38:8	821.5160	$C_{44}H_{74}N_2O_{10}P$	821.5076	10.2	9.5
40:1	863.6399	$C_{46}H_{92}N_2O_{10}P$	863.6485	10.0	2.5
40:9	847.514	$C_{46}H_{76}N_2O_{10}P$	847.5232	10.8	10.5
42:4	885.6416	$C_{48}H_{90}N_2O_{10}P$	885.6328	9.9	5.5
44:9	903.5950	$C_{50}H_{84}N_2O_{10}P$	903.5859	10.1	10.5
44:10	901.5800	$C_{50}H_{82}N_2O_{10}P$	901.5702	10.8	11.5
**GLYCEROLIPIDS**					
**TAG**					
45:2	778.6974	$C_{48}H_{92}NO_6$	778.6919	7.1	3.5
46:1	794.7294	$C_{49}H_{96}NO_6$	794.7232	7.8	2.5
46:2	792.7143	$C_{49}H_{94}NO_6$	792.7076	8.5	3.5
47:1	808.7431	$C_{50}H_{98}NO_6$	808.7389	5.2	2.5
47:2	806.7280	$C_{50}H_{96}NO_6$	806.7233	5.8	3.5
47:3	804.7125	$C_{50}H_{94}NO_6$	804.7076	6.1	4.5
48:1	822.7606	$C_{51}H_{100}NO_6$	822.7545	7.4	2.5
48:2	820.7442	$C_{51}H_{98}NO_6$	820.7389	6.5	3.5
48:3	818.7262	$C_{51}H_{96}NO_6$	818.7232	3.7	4.5
48:4	816.7102	$C_{51}H_{94}NO_6$	816.7076	3.2	5.5
48:8	812.6800	$C_{51}H_{90}NO_6$	812.6763	4.6	9.5
49:3	832.7365	$C_{52}H_{98}NO_6$	832.7389	-2.9	4.5
50:1	850.7940	$C_{53}H_{104}NO_6$	850.7858	9.6	2.5
50:2	848.7766	$C_{53}H_{102}NO_6$	848.7702	7.5	3.5
50:3	846.7574	$C_{53}H_{100}NO_6$	846.7545	3.4	4.5
50:4	844.7467	$C_{53}H_{98}NO_6$	844.7389	9.2	5.5
50:5	842.7294	$C_{53}H_{96}NO_6$	842.7232	7.4	6.5
51:1	864.8091	$C_{54}H_{106}NO_6$	864.8015	8.8	2.5
51:2	862.7926	$C_{54}H_{104}NO_6$	862.7864	7.2	3.5
51:3	860.7731	$C_{54}H_{102}NO_6$	860.7702	3.4	4.5
51:4	858.7611	$C_{54}H_{100}NO_6$	858.7546	7.6	5.5
52:1	878.8242	$C_{55}H_{108}NO_6$	878.8171	8.1	2.5
52:2	876.8089	$C_{55}H_{106}NO_6$	876.8015	8.4	3.5
52:3	874.7910	$C_{55}H_{104}NO_6$	874.7858	5.9	4.5
52:4	872.7783	$C_{55}H_{102}NO_6$	872.7702	9.3	5.5
52:6	868.7460	$C_{55}H_{98}NO_6$	868.7389	8.2	7.5
53:1	892.8400	$C_{56}H_{110}NO_6$	892.8328	8.1	2.5
53:2	890.8250	$C_{56}H_{108}NO_6$	890.8172	8.8	3.5
53:3	888.8049	$C_{56}H_{106}NO_6$	888.8015	3.8	4.5
53:4	886.7896	$C_{56}H_{104}NO_6$	886.7859	4.2	5.5
53:5	884.7771	$C_{56}H_{102}NO_6$	884.7702	7.8	6.5
54:2	904.8401	$C_{57}H_{110}NO_6$	904.8328	8.0	3.5
54:3	902.8207	$C_{57}H_{108}NO_6$	902.8171	4.0	4.5

	54:4	900.8074	$C_{57}H_{106}NO_6$	900.8015	6.5	5.5
	54:5	898.7938	$C_{57}H_{104}NO_6$	898.7858	8.9	6.5
	54:6	896.7789	$C_{57}H_{102}NO_6$	896.7702	9.7	7.5
	56:4	928.8354	$C_{59}H_{110}NO_6$	928.8328	2.8	5.5
	56:5	926.8208	$C_{59}H_{108}NO_6$	926.8172	3.9	6.5
	56:6	924.8084	$C_{59}H_{106}NO_6$	924.8015	7.6	7.5
	56:7	922.7940	$C_{59}H_{104}NO_6$	922.7859	8.8	8.5
**DAG**						
	34:1	612.5569	$C_{37}H_{74}NO_5$	612.5562	1.1	1.5
	36:2	638.5718	$C_{39}H_{76}NO_5$	638.5718	0.0	2.5
	36:3	636.5604	$C_{39}H_{74}NO_5$	636.5562	6.6	3.5
	38:4	662.5718	$C_{41}H_{76}NO_5$	662.5718	0.0	4.5
